# Steric exclusion and protein conformation determine the localization of plasma membrane transporters

**DOI:** 10.1038/s41467-018-02864-2

**Published:** 2018-02-05

**Authors:** Frans Bianchi, Łukasz Syga, Gemma Moiset, Dian Spakman, Paul E. Schavemaker, Christiaan M. Punter, Anne-Bart Seinen, Antoine M. van Oijen, Andrew Robinson, Bert Poolman

**Affiliations:** 1 Groningen Biomolecular Sciences and Biotechnology Institute, University of Groningen, 9700AB Groningen, The Netherlands; 2 Zernike Institute for Advanced Materials, Nijenborgh 4, 9747AG Groningen, The Netherlands

## Abstract

The plasma membrane (PM) of *Saccharomyces cerevisiae* contains membrane compartments, MCC/eisosomes and MCPs, named after the protein residents Can1 and Pma1, respectively. Using high-resolution fluorescence microscopy techniques we show that Can1 and the homologous transporter Lyp1 are able to diffuse into the MCC/eisosomes, where a limited number of proteins are conditionally trapped at the (outer) edge of the compartment. Upon addition of substrate, the immobilized proteins diffuse away from the MCC/eisosomes, presumably after taking a different conformation in the substrate-bound state. Our data indicate that the mobile fraction of all integral plasma membrane proteins tested shows extremely slow Brownian diffusion through most of the PM. We also show that proteins with large cytoplasmic domains, such as Pma1 and synthetic chimera of Can1 and Lyp1, are excluded from the MCC/eisosomes. We hypothesize that the distinct localization patterns found for these integral membrane proteins in *S. cerevisiae* arises from a combination of slow lateral diffusion, steric exclusion, and conditional trapping in membrane compartments.

## Abstract (web summary)

The yeast plasma membrane consists of membrane microdomains with distinct protein composition. Here the authors use high-resolution single molecule imaging to observe diffusion of specific transmembrane proteins in and out of these microdomains, and propose features that dictate their inclusion/exclusion from these structures.

## Introduction

The existence of compartmentalization allows cells to carry out specific functions at discrete locations in the cell or cellular membranes, which is one of the hallmarks of eukaryotic cells. Eukaryotic cell membranes contain hundreds of different lipids. In the plasma membrane (PM), these lipids are distributed asymmetrically over the two leaflets of the bilayer^1^. In mammalian cells, the PM has been shown to partition into small compartments, where proteins and lipids diffuse relatively quickly at short-distance scales, but in which long-range mobility is hindered by the membrane skeleton^2,3^. In this model, the hopping of molecules between compartments is a determining factor for the overall lateral motion. The existence of a membrane skeleton in the yeast *Saccharomyces cerevisiae* has not been demonstrated. However, its PM does contain discrete domains such as the membrane compartment occupied by Can1 (MCC) and the membrane compartment occupied by Pma1 (MCP)^4^. A protein scaffolding complex called the eisosome is located directly beneath the MCCs^5^; hence the name MCC/eisosomes. A yeast cell contains 30–50 such MCC/eisosome structures, which occupy 3–5% of the PM surface. The MCCs are enriched in ergosterol^6^, whereas the MCPs are rich in sphingolipids^7^. The functional role of the MCC/eisosome structures is not clear. They have been implicated in the protection of proteins from endocytosis, protein turnover, and protection to osmotic and other stresses^8–10^, but evidence is limited and sometimes controversial. Alternatively, the MCC/eisosomes may regulate the activity of transporters and other membrane proteins by providing a specific lipid environment. To better understand the function of MCC/eisosomes, it will be important to determine protein dynamics and partitioning in MCCs, MCPs, and possibly other domains.

The lateral motion of PM proteins in *S. cerevisiae* has been reported to be slow. However, it is not clear whether this slow diffusion arises from physical partitioning of proteins into microcompartments^11–13^ or from the physicochemical properties of the membrane itself. Here we show for solute transporters of similar size that the diffusion coefficient in the PM of *S. cerevisiae* is orders of magnitude lower than in the vacuolar membrane. To better understand the partitioning of proteins in the PM of *S. cerevisiae*, we performed dual-color super-resolution microscopy to (co)-localize proteins with the eisosomal marker, Pil1, and measured distance-dependent correlations in the locations of protein pairs in living cells. Additionally, we performed single-particle tracking (SPT) in combination with photo-activated localization microscopy (PALM) in total internal reflection fluorescence (TIRF) microscopy mode to determine the movement of proteins at the membrane plane of the cell relative to MCC/eisosomes. Our high-resolution microscopy analysis of the location and diffusion of a range of membrane proteins provides a new perspective on the structure and dynamics of the MCC/eisosome and the PM of yeast.

## Results

### High-resolution imaging of MCC/eisosomes

**Fig. 1:**
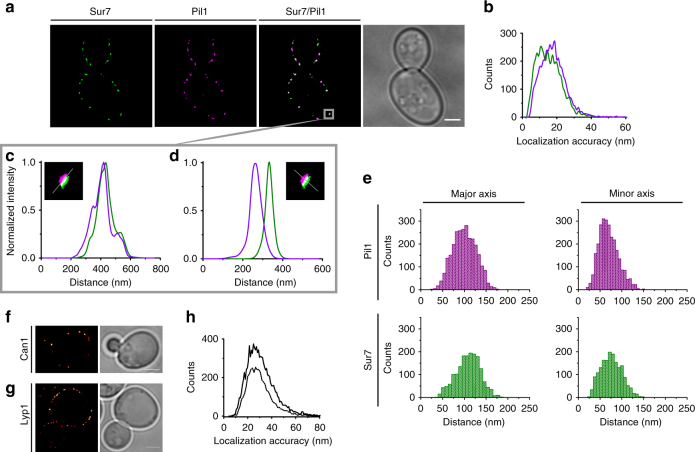
High-resolution plasma membrane protein localization. **a** Dual-color super-resolution reconstructions of Sur7-YPet in green and Pil1-mKate2 in magenta. Co-localizations appear in white. **b** The localization accuracy of the fluorophores YPet (green) and mKate2 (magenta) were estimated from the fitting error. **c**, **d** Eisosome line scans measured along (**c**) and perpendicular (**d**) to the plasma membrane. **e** Histograms of the distribution of the size of eisosomes on the basis of localizations of Pil1 or Sur7 (*n* = 302). Single-color super-resolution reconstructions of **f** Can1-mEos3.1 and **g** Lyp1-mEos3.1 with **h** their respective fitting errors (drawn line, Lyp1; dotted line, Can1). All proteins were chromosomally tagged with the respective fluorophores. Scale bar represents 2 µm; *n* represents the number of cells analyzed

We used dual-color super-resolution microscopy to study the localization of two MCC/eisosome-resident proteins, the integral membrane protein Sur7 and the scaffolding protein Pil1. We used the fluorescent proteins YPet and mKate2 as markers for these proteins, and carried out two-color imaging with the fusions expressed at endogenous levels. Importantly, there is no significant cross-contamination of signals arising from YPet and mKate2 in our setup. We find strong co-localization between Sur7 and Pil1 in our super-resolution reconstructions (Fig. 1a), which have a localization precision of about 20 nm for both YPet and mKate2 (Fig. 1b). The localization precision was taken from the fitting error of single molecules. Remarkably, we were able to resolve the membrane-indented structure of the MCC/eisosome and found that Pil1 is located slightly inside the PM. A magnified image of an MCC/eisosome with line scans along and perpendicular to the PM is shown (Fig. 1c, d, respectively; other examples are shown in Supplementary Figure 1). Our high-resolution images reveal that Sur7 and Pil1 are in fact spatially distinguishable from each other, with Pil1 being inset from the PM by 60 nm on average. We interpret this distance as reflecting the position of Sur7 at the edges of the MCC/eisosomal membrane and the soluble protein Pil1 forming the scaffold at the base of the MCC/eisosome.

Next, we estimated the dimensions of the MCC/eisosomes from the super-resolution data by determining the major and minor axis of the membrane compartments, which were obtained from the *x* and *y* coordinates of the localizations of Sur7-YPet and Pil1-mKate. Specifically, we determined the smallest ellipse containing a certain percentage of all localizations, which were obtained by analyzing the molecules at the bottom of the cells by PALM in TIRF mode. The dimensions in the plane of the membrane are comparable for the MCC and eisosomal marker; the average values taken from (Fig. 1e) are 109 ± 27 by 76 ± 24 nm for Sur7-YPet and 101 ± 26 by 71 ± 22 nm for Pil1-mKate (mean ± SD). These values are somewhat smaller than those determined by freeze-fracture electron microscopy in fixed cells^14^.

Analysis of the localization of the MCC protein Can1 by super-resolution microscopy, using the photo-switchable fluorescent protein mEos3.1 as fusion partner, shows a heterogeneous distribution in the PM (Fig. 1f), as one would expect for a protein associated with particular domain structures^4,5,10,15,16^. We find a similar distribution for Lyp1 (Fig. 1g), a sequence homolog of Can1 that has not been reported to reside in distinct membrane domains. Both proteins were expressed from their native chromosomal locus, under the control of their natural promoters. The localization precision, taken from the error of fitting single molecules, was ~30 nm (Fig. 1h). Inspection of the intensity fluctuations within the original microscopy movies indicates that the patches in the reconstructions are often composed of single molecules that are repeatedly localized in our analysis, as opposed to clusters of Lyp1 or Can1. Our data indicate that the endogenous levels of those proteins in the PM are relatively low; on the order of a few hundred molecules per cell, taking the photo-switching efficiency and other factors of quantitative PALM into account^17,18^. The low endogenous levels of Lyp1 and Can1 suggest that besides the allegedly MCC/eisosome partitioning of Can1, the proteins cannot form a smooth distribution as the number of molecules is not large enough.

### Cross-correlation of PM and eisosomal protein signals

**Fig. 2:**
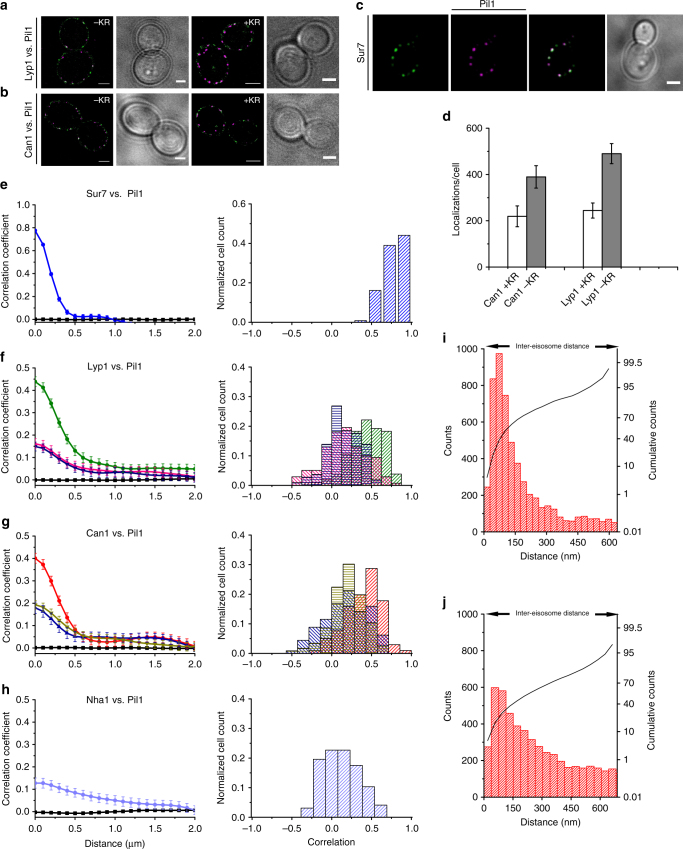
Substrate-dependent localization of proteins. Dual-color reconstructions of **a** Lyp1-L-YPet/Pil1-mKate2 and **b** Can1-L-YPet/Pil-mKate2 with and without lysine plus arginine in the growth medium, indicated as +KR and −KR, respectively. Wide-field images are depicted for clarity. All the scale bars represent 2 µm. **c** Cross-correlation of Pil1-mKate2 and Sur7-YPet. Panels: images were treated with a discoidal-averaging filter to better illustrate the localizations; the co-localization analysis was done with the raw diffraction-limited images. Wide-field images are depicted for clarity. **d** Number of localizations per cell of Lyp1 and Can1 with and without lysine plus arginine with error bars representing the standard deviation. **e**–**h** show cross-correlation of Pil1-mKate2 vs. proteins tagged with L-YPet; the left graph of each panel shows the correlation coefficients over distance for the various proteins with error bars representing standard error of the mean; the right graph of each panel shows the histograms of the probability distributions of single-cell cross-correlations. **e** Sur7 (blue; *n* = 118); **f** Lyp1 before addition of lysine plus arginine (green; *n* = 104), 40 min after the addition of lysine plus arginine (magenta; *n* = 138), and 120 min after the addition (blue; *n* = 108); **g** Can1 before addition of lysine plus arginine (red; *n* = 101), 40 min after the addition of lysine plus arginine (blue; *n* = 113) and 120 min after the addition (tan; *n* = 116); **h** Nha1 (light blue; *n* = 69). **i**, **j** Histograms showing the distance of Can1 molecules to the closest eisosome. Black lines indicate probability of finding an eisosomes at a discrete distance. **i** Can1 without arginine (*n* = 35); **j** Can1 with arginine (*n* = 47); *n* represents number of cells analyzed

We next carried out dual-color super-resolution microscopy to study the localization of Lyp1 and Can1 relative to the position of MCC/eisosomes at higher resolution than was available in previous studies^5,6,10,16^. Lyp1 and Can1 tagged with YPet partially co-localize with Pil1-mKate, both in the presence and absence of their substrates, lysine and arginine (Fig. 2a, b; control in Fig. 2c). Modulating the amount of lysine and arginine in the medium enabled control over the levels of each transporter in the PM (Fig. 2d). We quantified the co-localization between Lyp1 or Can1 and Pil1, using Van Steensel’s cross-correlation approach^19^. In this analysis, we use pairs of diffraction-limited images and measured line scans of fluorescence intensity along the PM and calculated the cross-correlation function between the two line scans to obtain information on co-localization. As a control, we first measured the co-localization of Sur7 and Pil1 (Fig. 2c, e). For this pair, a high correlation coefficient was observed at short distances (<200 nm; Fig. 2e), corresponding to strong co-localization of peaks of diffraction-limited size (MCC/eisosomes are smaller than the diffraction limit of the microscope (Figs. 1 and 2c). Both Lyp1 and Can1 show significant correlation with Pil1 in the absence of substrate (Fig. 2f, g; Supplementary Figure 2a and b). For both proteins, the correlation decreased rapidly with the addition of substrate and the total fluorescence decreased as a consequence of fast removal of the proteins from the membrane^20,21^. In the presence of substrate, the level of co-localization of Lyp1 and Can1 with Pil1 is moving to that of the sodium/proton antiporter Nha1, a membrane protein unrelated to Lyp1 or Can1, and not expected to be associated with MCC/eisosomes (Fig. 2h and Supplementary Figure 2c). The decrease in the short-distance cross-correlation features upon substrate addition and the decrease in fluorescence (Fig. 2f, g; Supplementary Figure 2a and b) suggests that Can1 (and possibly Lyp1) rapidly move out of the MCC/eisosome area and are then removed from the PM. Most likely, the conformational change upon substrate binding lowers the affinity of Can1 (and Lyp1) for a component in or near the MCC/eisosomes.

We further confirmed the substrate-dependent partitioning of Can1 using single-particle localization experiments in TIRF mode combined with high-resolution PALM imaging of the MCC/eisosomes. For this, we fused Can1 to mCardinal, a more photo-stable fluorophore, allowing for localizing single particles at the bottom of the cell. To determine the centroid (geometric center in the plane of the membrane) of the MCC/eisosome, we localized Sur7-YPet. Cross-correlation of trajectories of Can1 to the centroids of MCC/eisosomes (see Methods section) confirms the co-localization, as the peak of Can1 counts is found at 75 nm from the centroid of MCC/eisosomes (Fig. 2i). Experiments where the substrate was added 10 min prior to imaging confirm the movement of Can1 away from the MCC/eisosomes (Fig. 2j). We next tested whether partitioning of Can1 in the MCC/eisosome is proton-motive force-dependent as its dissipation by the protonophore FCCP has been claimed to cause a fast release of Can1 from the MCC/eisosome^6^). We repeated this experiment and found similar localization patterns for Can1 (and Lyp1) in the absence and presence of FCCP, albeit with a slightly higher distance correlation when the (electro)chemical proton gradient is dissipated (Supplementary Figure 3). Thus, unlike the substrate, the proton-motive force appears to play little or no role in the PM distribution of Can1 and Lyp1.

### Diffusion of proteins in the PM is very slow

**Fig. 3:**
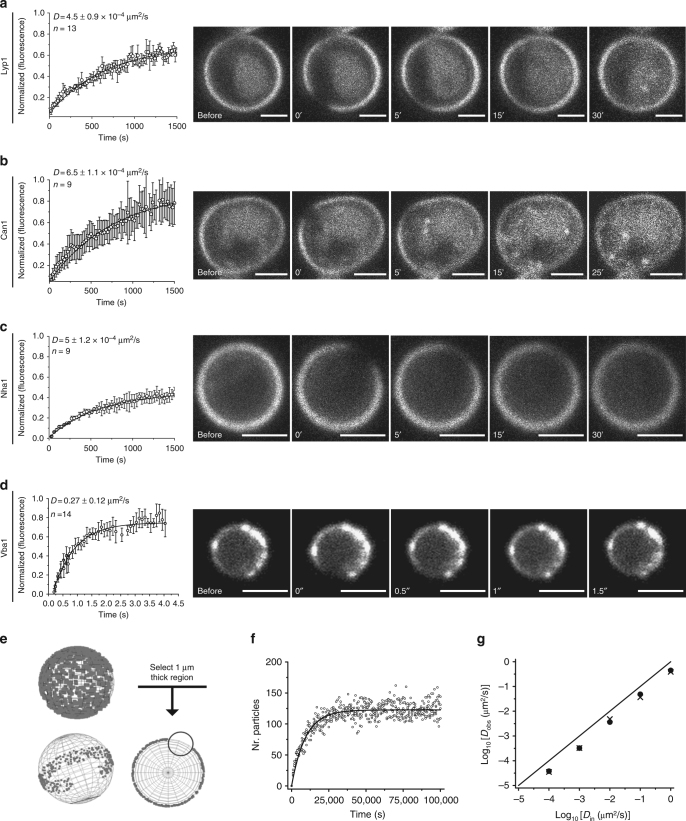
FRAP measurements to probe long-range diffusion. Normalized fluorescence recovery of YPet-tagged transporters expressed from a plasmid in the respective endogenous knockout strain: Lyp1-YPet (immobile fraction: 0.35) (**a**), Can1-YPet (immobile fraction: 0.15; *n* = 9) (**b**), Nha1-YPet (immobile fraction: 0.55; *n* = 9) (**c**), and Vba1-YPet (immobile fraction: 0.10; *n* = 14) (**d**). Confocal images of cells before and after photobleaching at different time points are shown in the right panels. Scale bars represent 2 µm; standard deviations and number of cells analyzed (*n*) are given in the graphs. **e** Spherical cell model used for simulation of Brownian diffusion as observed in a FRAP experiment. Photo-bleached region of 2 µm width and 1 µm thick. **f** Recovery of the particles in the bleached region (empty dots) and exponential fitting of the data (black line) are shown. **g** Comparison of input with observed diffusion coefficients for FRAP simulations. Every point indicates a separate simulation. The width and height of the bleached region are 2 and 2 µm, respectively. The black line represents the function *x*=*y*. All proteins were under overexpressed conditions; *n* represents the number of cells and error bars represent the standard deviation

The cross-correlation experiments show a relatively rapid removal of Lyp1 and Can1 from the MCC/eisosome after the addition of substrate. However, diffusion of integral membrane proteins has been reported to be very slow^11,13,22^. Exploring the idea of slow lateral diffusion, we determined the lateral diffusion coefficient of PM proteins using fluorescence recovery after photobleaching (FRAP) and SPT. For FRAP, the overexpressed membrane proteins were fused to YPet, and the diffusion of proteins in the PM was compared with that of a vacuolar membrane protein of similar size, Vba1. After photobleaching, Lyp1, Can1, and Nha1 showed similar recovery profiles and a single mobile fraction (Fig. 3a–c). The diffusion coefficients *D* of the PM proteins fall in the range of 4.5–6.0 × 10^−4^ µm^2^/s. The diffusion coefficient of the vacuolar solute/H^+^ antiporter Vba1 is 3 orders of magnitude higher (*D* = 0.27 ± 0.12 μm^2^/s; Fig. 3d) and similar to those previously measured for ER and other vacuolar membrane proteins^11,23,24^. In our FRAP measurements we analyzed the middle of yeast cells with molecules diffusing on a curved plane that we observe from the side. As the analysis is based on 2D diffusion, we investigated the accuracy of the analysis method. To this end, we simulated various FRAP experiments (Fig. 3e), analyzed the simulation results in the same way as the real data (Fig. 3f), and compared input with “observed” diffusion coefficients (Fig. 3g). The simulations show that the observed diffusion coefficients hardly deviate from the actual diffusion coefficient, validating our analysis method. Overall, the diffusion of the yeast PM proteins as probed by FRAP is remarkably slow and very different from the mobility of proteins in the PM of mammalian cells or the yeast organelles^3,25,26^. Consistent with the cross-correlation of Lyp1 and Can1 (Fig. 2f, g) with the MCC/eisosomes, an immobile fraction of Lyp1 and Can1 is observed and this fraction decreases when the expression of the proteins is increased. Overexpression of Lyp1 and Can1 leads to a smooth PM distribution (Fig. 3a, b), and only a small fraction of the total population is immobile under these conditions. These results suggest that the MCC/eisosomes have a limited number of sites for immobilizing membrane proteins.

### SPT shows conditional confinement of Can1 in the MCC

FRAP probes long-range diffusion of molecules and does not resolve barriers to short-range diffusion, such as confinement within specific membrane domains. Furthermore, the technique is limited to a relatively large number of molecules to redistribute, hence the need for protein overexpression. In order to resolve if Can1 partitions in the MCC/eisosomes, we combined PALM of Sur7-YPet with single-particle tracking of either Can1, Nha1, or Pma1. Critical for these measurements is the immobilization of cells. We found that classical coating techniques based on poly-l-lysine and concanavalin A^27,28^ are inappropriate for TIRF due to residual movement of the cells and background fluorescence, respectively. We therefore devised a new coating technique based on APTES-glutaraldehyde treatment of the glass slides, and we obtained excellent immobilization of *S. cerevisiae* with minimal background fluorescence (see Methods section). As photo-stability is a prerequisite for particle tracking, we fused Can1, Nha1, and Pma1 to mCardinal and followed the 2D diffusion of foci in the PM in TIRF mode.

**Fig. 4:**
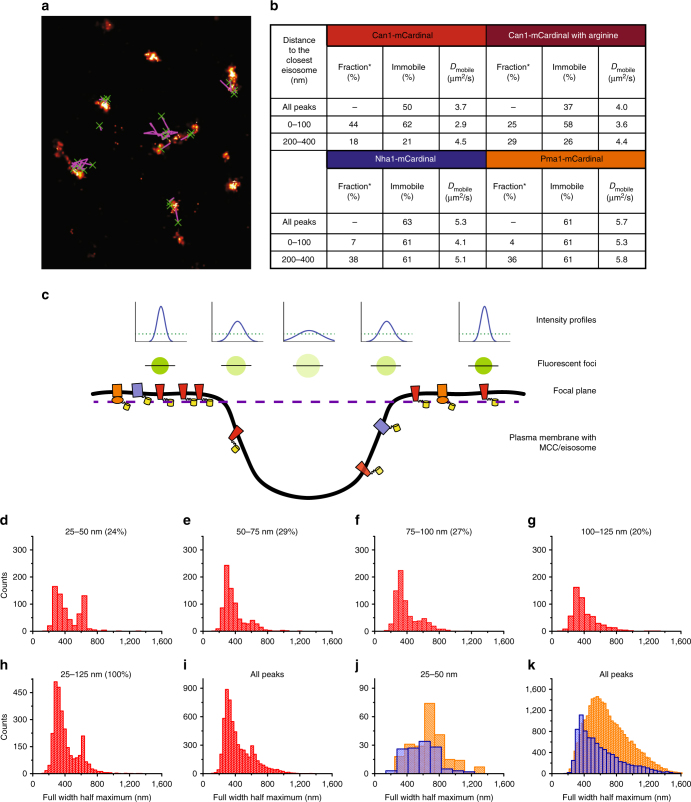
Lateral diffusion and distance dependence of membrane proteins relative to MCC/eisosomes. **a** Reconstruction of the trajectories: bright areas correspond to eisosomes, green Xs mark starting point of Can1 trajectory and purple lines show the trajectories. **b** Table summarizing diffusive behavior of Can1 (with arginine (*n* = 47) and without arginine (*n* = 35) in the medium), Nha1 (*n* = 52), and Pma1 (*n* = 129). *refers to fraction of peaks localized in the inter-eisosomal distance. **c** Cartoon showing the location of Can1 (red), Nha1 (light blue), and Pma1 (orange) relative to a MCC/eisosome and the intensity profiles of the fluorescent foci. The further away from the focal plane, the wider and dimmer the signal, which is seen in the intensity profiles of the peaks (green dotted lines indicate detection limit). Measuring the full width at half maximum (FWHM) of the peaks gives information about focal depth and thus the position of proteins in the MCC/eisosome; the extra peak at FWHM of 650 nm in panel **d** indicates that Can1 enters the MCC/eisosome in the z-direction. The panels **d**–**j** show the histograms of FWHM of Can1 of peaks detected at 25–50 nm (**d**), 50–75 nm (**e**), 75–100 nm (**f**), 100–125 nm (**g**), or 25–125 nm (**h**) from the centroid of the nearest MCC/eisosome; the percentages indicate the fraction of proteins at a given distance. The intensity profiles at 0–25 nm were too low to assign them confidently to Can1; the histogram of all Can1 peaks is shown in panel **i**. The panels **j** and **k** show the histograms of FWHM of Pma1 (orange) and Nha1 (light blue) at 25–50 nm from the centroid of the nearest MCC/eisosome (**j**) and of all the peaks (**k**); *n* represents number of cells analyzed

Tracking of Can1 molecules (Fig. 4a) in the PM and employing the cumulative probability distribution (CPD) analysis of its step sizes (see Methods section), we find that, at chromosomal levels of expression, about 50% of the population is mobile and has a diffusion coefficient of 3.7 × 10^−4^ μm^2^/s (Fig. 4b). These values are similar to those obtained by FRAP (Fig. 3b). In the FRAP experiments however, we biased Can1 to the MCP of the PM due to the unavoidable overexpression. We therefore determined the mobility of Can1 as a function of distance from the MCC/eisosomes. Within 200–400 nm from the centroid of an eisosome, 21% of the tracked Can1 molecules is immobile (our experimental limit to quantify mobility is around 10^−5^ μm^2^/s) (Fig. 4b; Supplementary Figure 4a, right panel), which is in agreement with the FRAP data (15%, see Fig. 3b). Importantly, the immobile fraction of Can1 increases toward the centroid of the MCC/eisosome. At a distance of 0–100 nm (mostly MCC/eisosome area), 62% of the Can1 molecules are immobile (Fig. 4b; Supplementary Figure 4a, left panel), indicating that a fraction of Can1 is trapped in the MCC/eisosomes.

The majority of Nha1 and Pma1 appear at a distance of around 300–400 nm, the region of the PM exactly in between two MCC/eisosomes; only 7% of Nha1 and 4% of Pma1 is found within 100 nm from the centroid of an MCC/eisosome (Fig. 4b and Supplementary Figure 4b, c). The diffusion of both Pma1 and Nha1 is not influenced by their proximity to MCC/eisosomes (Fig. 4b and Supplementary Figure 4b, c). Thus, we propose that Can1 (and Lyp1) diffuse in and out of MCC/eisosome area and a fraction of the molecules get trapped; Pma1 is excluded from MCC/eisosomes, and Nha1 may or may not enter but does not get trapped. Even though diffusion in the PM is slow, the rate is fast enough to allow proteins, inserted randomly, to reach an MCC/eisosome within 10 min.

Diffusion of a protein in the *z*-axis of the PM, that is the indentation of the MCC/eisosome, will result in out-of-focus movement and therefore results in detection of peaks with larger full width half maxima (FWHM) (Fig. 4c) and lower apparent diffusion coefficients. We observe this for Can1 (Fig. 4d–i) and find a population of Can1 with larger FWHM exclusively in the area of 25–50 nm around the centroid of the MCC/eisosomes (Fig. 4d). Such a population is not observed when the histograms of FWHM of Pma1 at 25–50 nm are compared with all peaks (Fig. 4j, k), indicating that Pma1 does not enter the MCC/eisosomes; in case of Nha1 a small shift toward larger FWHM values is observed (Fig. 4j, k) in agreement with the co-localization data (Fig. 2h), which suggests that Nha1 distributes more or less homogenously over the PM and can freely enter and leave the MCC/eisosomes. These data together with the distribution shown in (Fig. 2i) indicate that Can1 is indeed capable of diffusing into the MCC/eisosomes (25–50 nm from the centroid), but remarkably the majority of the molecules (76%) accumulate at a distance of 50–125 nm (referred to as outer edge of the MCC/eisosome area).

Upon addition of substrate, we see a shift of the Can1 population from the MCC/eisosome areas to MCP (Fig. 2i, j). Importantly, with substrate we also observe a decrease in the fraction of immobile Can1 (Fig. 4b). Thus, the correlation data (Fig. 2g) and the FWHM distributions of Can1 (Figs. 4d–i and 2i, j) suggest that without substrate a fraction of Can1 reaches the MCC/eisosome area and part of the molecules get trapped. In the presence of substrate, the distance correlation of Can1 (and Lyp1) to the MCC/eisosome decreases and the fraction of immobile Can1 decreases, which we take as strong evidence for release of proteins from the MCC/eisosome areas following a substrate-dependent conformational change (e.g., from inside-facing to outside-facing or vice versa^29–31^).

### Cytosolic domains hinder MCC/eisosome partitioning

**Fig. 5:**
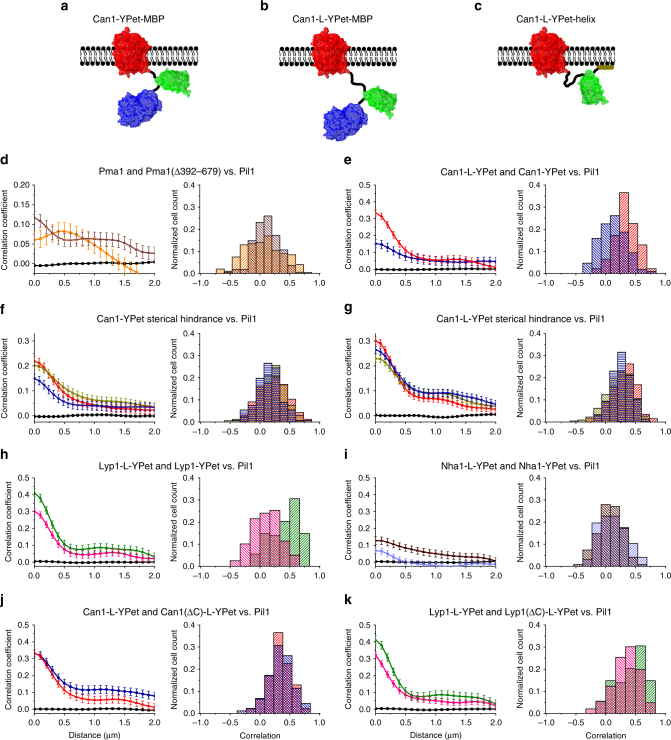
Steric occlusion from MCC/eisosomes. Schematic of transporter constructs (**a**–**c**) to investigate the possible hindrance for MCC/eisosome entry by engineering cytoplasmic domains onto Can1. Maltose-binding protein (MalE) (blue) attached to **c** Can1-YPet and **d** Can-L-YPet, and **e** Can1-L-YPet tethered to the membrane via an amphipathic α-helix and lipid moiety; the helix corresponds to the C-terminal 51 amino acids of Gap1^33^; L is linker as described in the methods section. Cross-correlation analysis of BY4742 cells expressing Pil1-mKate2 together with **d** Pma1-YPet (orange; *n* = 201) or Pma1(∆392–679)-YPet (brown; *n* = 169); the Pma1 constructs were expressed from a single copy plasmid under the control of the *Pma1* promoter. Cross-correlation of chromosomally labeled Pil1-mKate2 vs. chromosomally YPet-tagged target protein: **e** Can1-L-YPet (red; *n* = 93) and Can1-YPet (blue; *n* = 92). **f** Can1-YPet (red; *n* = 165), Can1-YPet-MBP (blue; *n* = 147), Can1-YPet-Gap1C (tan; *n* = 172); **g** Can1-L-YPet (red; *n* = 202), Can1-L-YPet-MBP (blue; *n* = 152), Can1-L-YPet-Gap1C (tan; *n* = 226); **h** Lyp1-L-YPet (green; *n* = 108) and Lyp1-YPet (magenta; *n* = 119); **i** Nha1-L-YPet (light blue; *n* = 69) and Nha1-YPet (pink; *n* = 122); **j** Can1-L-YPet (red; *n* = 93) and Can1(∆C)-L-YPet (blue; *n* = 88); **k** Lyp1-L-YPet (green; *n* = 108) and Lyp1(∆C)-L-YPet (magenta; *n* = 146). The left graph of each panel shows the correlation coefficients over distance for the various proteins with error bars representing standard error of the mean; the right graph of each panel shows the histograms of the probability distributions of single-cell cross-correlations; *n* represents number of cells analyzed

Most PM proteins do not partition in MCC/eisosomes. As observed for Nha1, those proteins may stochastically enter and leave these membrane structures without being trapped. However, proteins like the P-type ATPase Pma1 are reported to be excluded from MCC/eisosomes^15,32^ (Fig. 4j, k and Supplementary Figure 4e). In contrast to Lyp1, Can1 and Nha1, Pma1 contains a large cytoplasmic domain that may prohibit the protein from entering MCC/eisosomes. We tested the idea of steric hindrance by deleting the cytoplasmic domain of Pma1 and fusing cytoplasmic moieties to the C terminus of Can1 (Fig. 5a–c). When repeating our co-localization analysis for Pma1 and Pil1, we found a positive correlation at a distance of ~0.5 μm, corresponding to about half the distance between two MCC/eisosomes when measured half way the cell (Fig. 5d and Supplementary Figure 5). Indeed, deletion of the cytoplasmic domain of Pma1, resulting in Pma1(∆392–679), shows a positive correlation with a maximum at zero distance (Fig. 5d and Supplementary Figure 5), similar to what is seen for Nha1 (Fig. 2h).

In the measurements described thus far, all the fluorescent transporter constructs had a linker between the target protein and the fluorescent protein to provide flexibility. We then asked if the direct coupling of a fluorescent protein to a membrane protein could affect its localization, or its ability to enter MCC/eisosomes. We removed the 16-residue linker that connects YPet to the C terminus of Can1 and observed a significant decrease in the correlation of the protein with Pil1 (Fig. 5e; Supplementary Figure 5), which points toward exclusion by steric hindrance as a result of the tethering of a large, inflexible soluble domain. Next, we either increased the cytoplasmic body by fusing maltose-binding protein (MalE) to the C terminus of YPet (Fig. 5a, b) or tethered the YPet moiety more closely to the membrane surface (Fig. 5c) via an amphipathic α-helix with a lipid anchor^33^. Increasing the size of the cytoplasmic body (MBP linked to YPet) strongly affected the occlusion of Can1-YPet from MCC/eisosomes (Fig. 5f), whereas the tethering of the C terminus of YPet to the PM had little effect; however, the membrane anchor diminished a little the effect of the linker in Can1-L-YPet (Fig. 5g). We also find that the linker between Can1 and YPet reduces the hindrance effect of the extra protein domain, which is in accordance with an increased flexibility of the cytoplasmic domain relative to the membrane domain, giving the protein more degrees of freedom to move in and out of the MCC/eisosomes (Fig. 5g). In a similar approach, removal of the 16-residue linker connecting YPet with Lyp1 also decreased the correlation with the MCC/eisosomes (Fig. 5h). Removal of the linker for Nha1 had little effect, which is expected for a protein that does not co-localize with the MCC/eisosome (Fig. 5i). Finally, we wondered whether the observed effects are due to the short tethering or related to the accessibility of a specific sequence in the C-terminal amphipathic tail, that is present in wild type Can1 and Lyp1. When these tails are fused to GFP, they give rise to a patchy association of the proteins to the PM^33^. However, removal of the last 10 amino acids of the C terminus of Can1 and Lyp1 had no effect on the localization of the proteins (Fig. 5j, k; Supplementary Figure 5). Overall, we conclude that steric hindrance is a mechanism that can lead to exclusion of PM proteins from MCC/eisosomes.

## Discussion

**Fig. 6:**
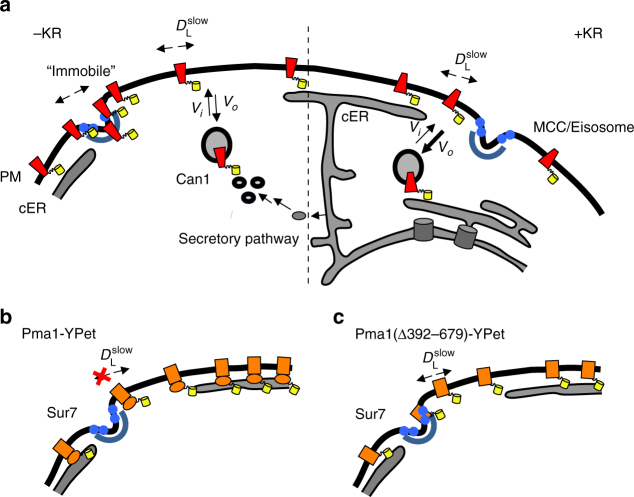
Cartoon summarizing the main findings on diffusion and localization of plasma membrane proteins. **a** The plasma membrane (PM), cortical ER (cER) and two MCC/eisosomes (Sur7 in the membrane and Pil1 scaffold) are shown. The scaffolding of the MCCs is shown as blue half circle (Pil1); the blue small circles depict Sur7*. D*_L_, *V*_*i*_ and *V*_*o*_ refer to lateral diffusion and the rate of exo- and endocytosis, respectively. Left: in the absence of substrate (−KR): a fraction of Can1 (red) accumulates in (near) the MCC/eisosomes and has an apparent *D*_L_ < 10^−5^ μm^2^/s, here indicated as “immobile”. The yellow cylinder depicts the fluorescent proteins fused to the transporters. The total concentration of Can1 is stable as delivery to (*V*_*i*_) and removal from (*V*_*o*_) the membrane are similar. Right: in the presence of substrate (+KR): Can1 takes a different conformation and dissociates from the MCC/eisosome and diffuses out. Next, Can1 is ubiquitinated and rapidly removed from the membrane (*V*_*o*_ > *V*_*i*_; indicated by thickness of arrow). **b** Large cytosolic domains exclude proteins from entering MCC/eisosomes, as shown for Pma1-YPet; **c** removal of the cytoplasmic domain enables (Pma1(∆392-679)-YPet) to enter the MCC/eisosome

Studying membrane protein dynamics and localization at the single-molecule level provides much more insight into their spatial organization and dynamics than is possible with conventional methods. We show that the physical confinement of Can1 takes place at the (outer) edge of the MCC/eisosomes and show removal of the transporter upon the addition of substrate; for Lyp1 we make very similar observations (Fig. 6a). The fraction of confined Can1 decreases with increasing expression level, suggesting that the MCC/eisosome area has a limited number of binding sites for the protein. Moreover, we show that proteins with large cytoplasmic domains closely spaced near the membrane surface (e.g., Pma1), and constructs with limited flexibility between the membrane domain and fluorescent reporter (fusions without linker and ending with an amphipathic helix), are excluded from MCC/eisosomes (Figs. 5 and 6b, c). Steric exclusion could be a more general factor in PM localization of proteins in budding yeast, and contribute to the domain formation as observed by Spira and coworkers, who claim that many proteins have their own independent “domain”^32^. Besides the MCC/eisosome, we did not observe such specific domains, however all PM proteins probed by SPT and FRAP showed a significant immobile fraction. Even a substantial fraction of Pma1 and Nha1, which are fairly homogeneously localized in the MCP area, is immobile (Fig. 4). The molecular basis for the immobility of these proteins warrants further investigation as it seems to have a different basis than in mammalian cells where the membrane skeleton hinders free diffusion^2^.

Transporters that are reported to partition in the MCC/eisosomes are Can1, Tat2 and Fur4^4,10,15^. We now also find a positive correlation of Lyp1 with the MCC/eisosome structures (Fig. 2f). It has been previously suggested that partitioning of these transporters in MCC/eisosomes is disrupted by the dissipation of the proton-motive force by the protonophore FCCP^6^. However, we could not confirm these findings.

MCC/eisosomes form small invaginations in the membrane caused by the BAR domain proteins Pil1 and Lsp1, that assemble into an elongated network of banana-shaped dimers and stimulate membrane curvature^34,35^. Pil1 and Lsp1 create a specific environment in the overlaying MCC, which is locally curved and increased in PI(4,5)P2 concentration^36^. We now confirm with high-resolution fluorescence microscopy in live cells the membrane-indented structure of the eisosome as previously observed with electron microscopy^14^ and show that the MCC (PM) area, marked by Sur7, and the (scaffolding) eisosome structure, marked by Pil1, have similar dimensions. Under native expression conditions without substrate, we find that Can1 is predominantly present on the (outer) edge of the MCC/eisosomes, which could be related to the high local curvature or the presence of specific binding partners. Importantly, the proteins readily dissociate from this area upon addition of substrate. It has been suggested that the MCC area is essential for the activity of Can1^32^, but this idea is at variance with the notion that substrate alters the conformation of Can1 and Lyp1^29,37^ and, as we find, removes the proteins from the MCC/eisosomes. Moreover, the localization of Can1 with MCC/eisosome markers decreases with increasing expression level whereas arginine and lysine transport activity increases. Finally, we have shown that purified Lyp1 is active in model membranes devoid of MCC/eisosomes^29^.

In conclusion, proteins in the yeast PM diffuse extremely slowly and a fraction of the proteins is (conditionally) immobile. The high fractions of sphingolipids with very long saturated acyl chain(s) and ergosterol^7^, and the overall highly ordered bilayer structure, may explain the slow diffusion and make yeast highly tolerant to adverse environmental conditions. We report MCC/eisosomes as barriers for diffusion, because membrane proteins with large cytosolic domains and proteins with little flexibility between the domains cannot enter the compartment. It is not entirely clear why Can1 (and Lyp1) are conditionally trapped in MCC/eisosomes and other proteins (e.g., Nha1, Pma1) are not. Steric hindrance clearly is a factor but is not the only determinant of membrane localization. We find that after stochastic insertion into the membrane and slow Brownian diffusion Can1 (and Lyp1) reach the MCC/eisosomes, where a fraction of the molecules bind conditionally at the (outer) edge of the MCC/eisosome structure. Addition of substrate changes the conformation of Can1 and Lyp1, which disrupts the interactions with the binding partner(s) and the proteins diffuse away from the MCC/eisosomes. Our data are compatible with the idea that MCC/eisosomes have limited storage capacity for proteins that are not active due to unavailability of substrate.

## Methods

### Growth conditions, plasmids, and strains

Yeast cells were grown for at least 72 h at 30 °C at 200 rpm. We used synthetic dropout media containing 2% [w/v] of carbon source: D-raffinose in strains containing constructs under the *gal* promoter and glucose in the other cases. For FRAP experiments, cells grown in glucose medium were transferred to medium containing both D-raffinose (2% [w/v]) and glucose (0.1% [w/v]). The cultures were diluted in the morning and afternoon to sustain growth in the exponential phase. Strains with fluorescent constructs of Can1, or Lyp1, were grown without arginine and lysine in the media, instead a lysine di-peptide was added to allow growth.

All strains are based on *S. cerevisiae* strain BY4742 (Supplementary Table 1). Genomic DNA isolation of *S. cerevisiae* BY4742 was carried out according to Sherman et al.^38^. For the amplification of DNA, using uracil containing primers, the polymerase chain reactions (PCRs) were performed with PfuX7^39^. Amplified fragments were assembled into full plasmids (Supplementary Table 2) by treatment with DNA glycosidase and DNA glycosylase-lyase endo VIII, commercially available as USER, following the manufacturer’s instruction (New England Biolabs, Ipswich, MA, USA). Ligation products were transformed into chemically competent *E. coli* MC1061 cells^40^. All constructs assembled from PCR fragments were verified by DNA sequencing. Genomic tagging and deletion of genes were done with standard PCR-based homologous recombination, using the primers listed in (Supplementary Table 3). Transformation of plasmids and linear constructs into *S. cerevisiae* was performed as described by Drew et al.^41^

The plasmids pFB001, pFB002, pFB003, pFB004, pfB005, and pFB006 were constructed by four-way ligations of PCR fragments, in which the backbone of the pRS426GAL1-GFP vector was amplified with primer pairs Pr1/Pr2 and Pr3/Pr4, two fragments that exclude the GFP coding region. The fragment coding for the *YPet* gene was amplified from a synthetically generated coding sequence ordered from (GeneArt, Regensburg, Germany), using primer pair Pr5/Pr6. The insert was amplified from *S. cerevisiae* BY4742 chromosomal DNA with primer pair: Pr7/Pr8. Similarly plasmids were constructed for *can1*, *nha1, and vba1* using primer pairs Pr9/Pr10, Pr11/Pr12, and Pr13/Pr14, respectively. The pLS006 plasmid was created by amplification of pUG73 vector with primer pair Pr62/63, and the *gap1C* fragment was amplified with Pr64/65 using pDP001-GFP-Gap1C as template. For the pLS003 and pLS004 plasmids, the backbone and the *ura3* marker were both separately amplified from the pFB001 plasmid using primer pairs Pr1/Pr3 and Pr2/Pr4, respectively. The inserts were amplified from the respective strain using primer pairs Pr51/53 and Pr52/53, respectively. All these plasmids were assembled by the uracil-excision method subsequently pieces were combined treated with USER enzyme, transformed in *E. coli*, and isolated.

The pFB007, pFB008, and pFB009 plasmids are based on three PCR fragments, using the uracil excision-based cloning method. The backbone and the *ura3* marker were both separately amplified from the pug72 plasmid using primer pairs Pr19/Pr22 and Pr20/Pr21, respectively. *mEos3.1*, *YPet*, or *mKate2* was amplified using primer pair Pr23/Pr24, Pr25/Pr26, or Pr27/28 from a synthetically generated coding sequence, ordered from (GeneArt, Regensburg, Germany). The fragments were treated with USER and transformed into *E. coli* MC1061 as described previously. For the construction of C-terminal fusion proteins on the chromosome, we made use of the *ura3* selection marker and the ability for its counter selection on 5 fluoro-orotic acid (5FOA) as described by Alani et al.^42^. For genomic tagging of *lyp1*, *can1*, *sur7* and *pil1* with either *mEos3.1 or YPet*, or *mKate2*, we amplified *mEos3.1*, *YPet* or mKate2*-ura3* cassette from pFB007, pFB008, and pFB009, respectively.

For the tagging of *can1, lyp1, nha1, pil1*, and *sur7* with/without linker or deletion of the sequence coding for the last 10 amino acids of Can1 and Lyp1, we used primer pairs Pr29/30, Pr34/35, Pr38/39–40, Pr41/Pr42, Pr43/Pr44, Pr29/33, and Pr34/37, respectively. The amplified cassettes were transformed into *S. cerevisiae* BY4742 and homologous recombination of the cassette into the genome was selected for by growth on a uracil-depleted medium. The Ura3 marker was removed from the chromosome by recombination of its homologous flanking regions, for which we selected for growth on a medium containing 5FOA. For the labeling of a second gene product in the same strain with a different fluorophore the above steps were repeated, except for the counter selection on 5FOA.

pLS001 is a derivative of pRS316 with *Pma1-YPet* integrated. *Pma1* with 848 bases upstream and YPet were PCR amplified using primer pair Pr45/46 and Pr47/Pr48, respectively. The pRS316 vector was digested with a blunt end cut using *Sma*I via manufacturers protocol (New England Biolabs). The complete vector was created via a three way homologous recombination in *S. cerevisiae*. pLS002 is a derivative of pLS001 in which *Pma1* was truncated by PCR amplification of pLS001 and using primer pair Pr49/Pr50 a circular vector was formed by homologous recombination of both ends.

To introduce an additional protein domain at the C-terminal end of YPet, we used the primer pair Pr58/59 for *gapC* (C terminus of Gap1) and pR60/61 for *malE*. The amplified cassettes were transformed into *S. cerevisiae* BY4742, and homologous recombination of the cassette into the genome was selected by growth on leucine- (in case of *gap1C*), or histidine- (in case of MBPs) depleted medium.

The pLS005 vector was constructed by amplifying the backbone of pLS003 with the primer pair Pr54/55. The fragment coding mCardinal was amplified with primer pair Pr56/57, using a synthetic codon-optimized mCardinal gene as template (GeneArt, Regensburg, Germany). The pLS007 was constructed by amplifying the backbone of pLS005 with two pairs of primers: Pr66/67 and Pr68/69. The DNA fragment coding for Nha1 was amplified from the genome of *S. cerevisiae* using primer pair Pr70/71 a circular vector was formed by homologous recombination of overlapping ends in *S.cerevisiae*. Similarly plasmid pLS008 was constructed by amplifying the backbone of pLS005 with two pairs of primers: Pr66/67 and Pr68/69. The DNA fragment coding for Pma1 was amplified from the genome of *S. cerevisiae* using primer pair Pr72/73.

### Microscopy equipment

For super-resolution microscopy measurements, a fully automated home-built microscope was used^43^. We constructed a wide-field single-molecule fluorescence microscope by coupling high-power laser excitation into a commercially available inverted fluorescence microscope body (IX-81, Olympus), equipped with a 1.49 NA ×100 objective and a 512 × 512 pixel EM-CCD camera (C9100-13, Hamamatsu). Excitation light was provided by continuous wave optically pumped semi-diode lasers (Sapphire LP, Coherent) of wavelength 514 nm (150 mW max. output) and 568 nm (200 mW max. output). For imaging mKate2 and mEos3.1 fusions, we used 568 nm excitation light and collected light emitted between 610 and 680 nm (ET 645/75 m filter, Chroma). For imaging YPet fusions, we used 514 nm laser excitation and collected light between 525 and 555 nm (ET540/30 m filter, Chroma). For FRAP measurements, a commercial laser-scanning confocal microscope, LSM 710 (Carl Zeiss MicroImaging, Jena, Germany) was used. The microscope was equipped with a C-Apochromat ×40/1.2 NA objective and a blue argon ion laser (488 nm). For the SPT experiments, a home-built Olympus IX-81-ZDC inverted TIRF microscope was used. The microscope was equipped with a UAPON 1.49 NA ×100 TIRF objective (Olympus, Inc), a manual open-frame microscope stage (M-545) (Physik instruments, Inc), a 512 × 512 Electron Multiplying Charge-Coupled Device (EMCCD) C9100-13 camera (pixel size 80 nm, EM gain 1200×) (Hamamatsu, Inc) and Xcellence^®^ software (Olympus, Inc). Fluorescent proteins were excited with continuous wave (CW) lasers (Coherent Sapphire, Inc). During the microscopy experiments, Z-drift was compensated by z-axis control, which is an option built into the IX81. Emission was filtered using bandpass filters obtained from AHF^®^ (AHF, Inc). For imaging Ypet, the bandpass filter HC535/22 (Semrock, Inc) was used, whereas for imaging mCardinal the bandpass filter HC630/92 (Semrock, Inc) was used.

### Fluorescence recovery after photobleaching measurements

All fluorescence recovery after photobleaching (FRAP) measurements were performed on cells expressing the target protein from plasmids pFB001, pFB002, pFB003, and pFB004 in their respective endogenous knockout strain. Cells were induced with 0.2% [w/v] galactose for 2.5 h prior to the FRAP measurement and subsequently suspended in glucose medium to avoid further transcription. Cells were immobilized in between two microscope slides and the focal plane positioned to the mid-section of the cells. Subsequently, an area, corresponding to the PM or vacuolar membrane (VM), with a radius of ~1.0 µm was photo-bleached with a short (26 µs) focused high-power light pulse. Immediately afterwards, several images of the fluorescence recovery were collected every 20 s or 110 ms for the plasma or vacuolar membrane, respectively, over a total time period of 2400 s and 5 s, using a laser output power of 517 W/cm^2^. During the entire experiment, the stage was heated to 30 °C, using a Pecon climate chamber. Data analysis was carried out in imageJ^44^. Images were corrected for *x*–*y* drift using cross-correlation fitting. The fluorescence intensity over time of the PM was corrected for photobleaching effects by fitting the decay to a single exponential. The bleaching area was selected and the recovery was fitted with Eq. (1) to find the half-time of recovery.

1 $$f\left( t \right) = A\left( {1 - \mathrm{e}^{\frac{{ - \ln \left( 2 \right)}}{{\tau _{0.5}}}t}} \right)$$

The diffusion coefficient (*D*) was estimated according to Eq. (2), derived from Axelrod et al.^45^:

2 $$D = \gamma \frac{{w^2}}{{4\tau_{0.5}}}$$

where *D* is the diffusion coefficient, *w* the radius of the bleaching spot, *τ*_0.5_ the half-time of recovery and *γ* a correction factor which is 0.88 for circular beams. The radius of the bleaching spot was 1.0 ± 0.1 μm as determined by Meinema et al.^23^.

The analysis methods for FRAP are designed to determine the diffusion of molecules in a plane. Here, we were looking at the middle of yeast cells with molecules diffusing on a curved plane that we observe from the side. We thus investigated the accuracy of the analysis methods by simulating the various experiments and comparing input with “observed” diffusion coefficients. All simulations were performed in Smoldyn^46^, which simulates particles undergoing random walks on specified geometries. Further analysis of simulated trajectories was performed in Mathematica. For the FRAP simulations we used a sphere with a radius of 2.5 µm. For each simulation, 5000 particles were distributed randomly over most of the surface, leaving a “bleached” area free of particles. Two bleach area sizes were used: (1) a (nearly) rectangular region of 2 µm in width and 1 µm in height and (2) a (nearly) square region of 2 µm in width and 2 µm in height. The width used here is similar to the width of the bleaching area in the experimental FRAP measurements. Five simulations were performed with the small rectangular bleach area. All with an input diffusion coefficient of 10^−4^ µm^2^/s, a simulation time step of 0.2 s and a total simulation time of 10^5^ s. Ten simulations were performed with the big square bleach area with five different diffusion coefficients, 10^−4^–1 µm^2^/s. The simulation time steps and total simulation times were 0.2–2 × 10^−5^ s and 10^5^–10 s, respectively (in steps of 10-fold). For each simulation, the number of particles in the bleached area was recorded over time. The recovery profile was fitted with Eq. (1). The obtained time constant, *τ*_0.5_, was used in Eq. (2) to calculate *D*.

### Single-particle localization analysis

Single-particle localization was performed by using custom-written plug-ins for ImageJ. Photon detection using the EMCCD camera results in point spread functions (PSFs), which can be modeled by a two-dimensional Gaussian function (Eq. 3). Here, *b* is the background pixel intensity, coefficient *A* is the amplitude, *x*_0_ and *y*_0_ correspond to the center position, *σ*_*x*_ and *σ*_*y*_ are the *x* and *y* spread of the PSF.

3 $$f\left( {x,y} \right) = b + A * \mathrm{e}^{ - \left( {\frac{{\left( {x - x_0} \right)^2}}{{2\sigma _x^2}} + \frac{{\left( {y - y_0} \right)^2}}{{2\sigma _y^2}}} \right)}$$

To detect all foci per frame, we applied a discoidal filter^47^ to reduce noise. Two images are generated, one by applying a discoidal-averaging filter with a diameter of 3 pixels, another one by applying an annular averaging filter with a width of 1 pixel and a diameter of 7 pixels on the original image. The difference between the two images is subsequently used to find local maxima. Pixels with a value 4–5 times the standard deviation above the mean pixel value were regarded as peaks. We fitted a two-dimensional Gaussian function (Eq. 3) to all peaks on the original non-filtered image using the Levenberg–Marquardt method^48,49^. The resulting Gaussian profiles gave the sub-pixel coordinates of the peak positions (corresponding to *x*_0_, *y*_0_ of Eq. 3) for each frame.

### Size determination of MCC/eisosomes

Cells expressing Sur7-YPet and/or Pil1-mKate2 were premixed with fluorescent microspheres (TransFluoSpheres (488/560)), then embedded in 0.5% (w/v) low-melting agarose and placed in between two microscope slides. Single fusion strains showed no bleed-through between channels. YPet and mKate2 are not known to be photo-switchable, but we successfully used the proteins for high-resolution imaging by first forcing the molecules into a dark state with an intense laser pulse (1800 W/cm^2^) at the excitation maximum of the fluorophore (514 or 568 nm) and, subsequently, re-activating individual molecules with a 405 nm laser and imaging with the excitation lasers. Typically, 1000 frames were recorded in each fluorescence channel, collected at room temperature (20 °C). Super-resolution image reconstructions were generated after processing of the data with home-written software for dual-color PALM. Single particles were localized with a localization accuracy of ~ 30 nm. We corrected for chromatic aberration using the fluorescent microspheres. The dimensions of the MCC/eisosome compartment were determined from the reconstructions and measured as shown (Fig. 1c, d and Supplementary Figure 1). All data was averaged, and it was taken into account that MCC/eisosomes were imaged randomly from different angles. We calculated width and length values that would match the averaged sampling value, under the assumption that all pictures were made with a random MCC/eisosome orientation.

### Super-resolution imaging of Can1 and Lyp1 molecules

Cells expressing Can1-mEos3.1, or Lyp1-mEos3.1 were premixed with fluorescent microspheres. For mEos3.1 imaging excitation light (*λ*_EX_ = 568 nm) was introduced at 180 W/cm^2^ for all the samples. A second laser (*λ*_EX_ = 405 nm) was used to photo-switch individual mEos3.1 molecules from a green to a red fluorescent state. The laser power was adjusted to activate only a small sub-set of molecules at a time and was kept the same for all the experiments. Typically, 5000 frames were collected per measurement, with the microscope at ~20 °C.

For the super-resolution dual-color imaging of Lyp1-Ypet and Can1-Ypet vs. Pil-mKate2 the same method was applied as described in the paragraph above. Reconstructions were made as shown in Fig. 2a, b. The total number of localizations of Can1-Ypet and Lyp1-Ypet at the middle of the cell was counted and averaged per cell in each medium condition shown in Fig. 2d.

### Cross-correlation microscopy

Cells expressing Pil1-mKate and one of the following constructs: Sur7-YPet, Lyp1-YPet, Lyp1-L-YPet, Lyp1(∆C)-L-YPet, Can1-YPet, Can1-L-YPet, Can1(∆C)-L-YPet, Can1-L-YPet-MBP, Can1-L-YPet-gapC, Can1-YPet-MBP, Can1-YPet-gapC, Nha1-YPet, Nha1-L-YPet, or with pLS001, or pLS002 plasmids were grown to early exponential phase (OD of 0.1–0.3) and condensed by centrifugation at 3000×*g* for 5 min. In the experiment testing effect of substrate on localization of the protein, arginine and lysine (1.25 mM) was added 40, or 120 min before the centrifugation. Cells were immobilized in between two microscope slides and the focal plane positioned to the mid-section of the cells. We imaged mKate2 with excitation from the 568 nm laser (75 W/cm^2^) with 30.5 ms exposure time and YPet with excitation of 514 nm laser (75–180 W/cm^2^, depending on protein imaged) with 30.5 ms exposure time. Co-localization analysis of line-scan data was performed using van Steensel’s approach^19^. To generate line scans, a 500 nm wide line selection was drawn around the periphery of each cell in ImageJ^44^, and the fluorescence intensity in each of the two-color channels recorded as a function of position along the line. For each pair of YPet and mKate2 line scans, the Pearson correlation coefficient between the color channels was calculated using a home-written Python program, the intensity information of one line scan was shifted by one pixel unit (100 nm) and the correlation coefficient recalculated. This process of pixel shifting and correlation was repeated to produce plots of correlation coefficient vs. shift distance for each cell. The plots were then averaged to obtain a measure of intensity correlation vs. distance along the cell periphery. To measure the response of uncorrelated line scans, we paired each YPet line scan with a spatially unrelated mKate2 line scan, selected randomly from a different cell of the same strain. The random selection and analysis steps were repeated 100 times and the correlation coefficient vs. shift distance series of all cells were averaged.

### Super-resolution microscopy in TIRF mode and size of eisosomes

Prior to silanization with APTES, coverslips were cleaned from fluorescence impurities. First, high precision coverslips, 75 × 25 mm (0.17 mm thickness), were sonicated for 30 min at 30 °C in acetone (99.5%), and, subsequently, sonicated for 45 min at 30 °C in 5 M KOH. After sonication, the coverslips were washed several times with double-distilled H_2_O (ddH_2_0). Residual solvents were dried out using pressurized N_2_ and 30 min incubation at 110 °C. Next, the coverslips were plasma cleaned (PE-50, Plasma Etch, Inc) for 10 min. Directly after plasma cleaning, 2% APTES (v/v) in acetone was added to the coverslips. After 10 s, coverslips were washed several times with acetone and were dried out by using pressurized N_2._ The APTES coated coverslips were then stored under vacuum. Prior to the microscopy experiment, a bottomless µ-Slide (Ibidi, Inc) was placed on an APTES coated coverslip. In order to attach glutaraldehyde to the APTES, 5% glutaraldehyde was added to each well. After 10–15 min incubation, each well was washed several times with ddH_2_O before addition of cells. The procedure resulted in a covalent bond between the free aldehyde groups of the glutaraldehyde and the amino acid groups present on the cell wall surface of yeast cells^50^.

Cells expressing Pil-mKate2, and Sur7-Ypet were grown to mid-exponential growth phase (OD_600_ 0.5–0.7) were centrifuged at 3000×*g* for 4 min. The pellets were resuspended in ddH_2_O and mixed with TransFluoSpheres beads (488/560). Cells were subsequently added to wells of a bottomless µ-Slide (Ibidi, Inc) stuck onto an APTES-glutaraldehyde-coated coverslip (see above). Prior to imaging, low fluorescence medium lacking lysine and arginine was added to maintain cells in a healthy state during the microscopy experiments. Imaging of YPet was accomplished by 514-nm excitation with a power density of 1.4 kW/cm^2^. Movies of 600 frames were taken for each fluorescent channel. In order to excite mKate2, light of 561 nm was provided with a power density of 0.7 kW/cm^2^. Both for imaging YPet and mKate2 molecules an exposure time of 30.5 ms was used.

The dimensions of the eisosomes were determined by calculating the major and minor axis from all fitted peak positions. First, the positions of all eisosomes are determined by thresholding a super-resolution reconstruction of all fitted peaks. For each eisosome we consider all fitted peaks that have a distance of less than one pixel from its center. From the fitted peaks, that belong to the eisosome, we determine its orientation by using linear regression. The fitted peaks are subsequently rotated such that the major axis is along the *x*-axis and the minor axis along the *y*-axis. The length of the major and minor axis is determined by fitting an ellipse that contains 75% of all peaks. By taking a fraction of all peaks we filter out outliers. Including all fitted peaks would result in an overestimate of the major and minor axis.

### SPT in TIRF mode and distance correlation to MCC/eisosomes

Cells expressing Sur7-YPet and Can1-mCardinal, or carrying pLS005, pLS007, and pLS008, grown to mid-exponential growth phase (OD_600_ 0.5–0.7), were centrifuged at 3000×*g* for 4 min. Cells with plasmids were induced for 40 min by 0.1% D-galactose prior to centrifugation. Slides were coated and samples were prepared as described in section (Preparation of APTES-glutaraldehyde-coated coverslips for SPT experiments). A TIRF time-lapse movie was obtained by exciting with a 561 nm laser (0.5 kW/cm^2^), using an exposure time of 30.5 ms every 10 s for 150–200 frames. Subsequently, imaging of YPet molecules was accomplished by 514 nm excitation (1.4 kW/cm^2^), using an exposure time of 30.5 ms. In this way, YPet was forced into a short-lived photo-darkened state. After data collection, the time-lapse movies were drift-corrected by fitting the fluorescent beads with a two-dimensional Gaussian function; the beads were tracked throughout the movie. The offset between the two different channels was measured by the difference in fitted-position of all the fluorescent beads on the first frame of the two corresponding movies. By using the fitted coordinates of Sur7-YPet, we constructed a high-resolution image of the eisosomes. The resulting peaks in the reconstructed image were then clustered by iteratively applying a mean-filter with decreasing radii (400–120 nm). The precise center-coordinates of the eisosomes were determined by averaging all the centroids of the peaks that were located within radial distance of 240 nm from the maxima in the filtered high-resolution image.

SPT trajectories of Can1-mCardinal, Nha1-mCardinal, and Pma1-mCardinal were obtained from the TIRF time-lapse movies. These trajectories were constructed by linking together peaks that appear within a pre-defined radius of 240 nm in subsequent frames. The distance from the starting point of each trajectory to the center-coordinate of its closest eisosome was then calculated and plotted in an histogram using Eq. (4) for the optimal bin width^51^. Besides, for each eisosome, the closest distance to a neighboring eisosome was calculated.

4 $${\mathrm{Bin}}\,{\mathrm{width}} = 3.49\sigma n^{ - \frac{1}{3}}$$

Data analysis was exclusively performed within certain regions of interests (ROIs) that excluded edges of cells, to avoid membrane curvature. Heterogeneity in the diffusion behavior of the proteins was analyzed by the cumulative probability distribution (CPD) of the obtained step sizes. The CPD function gives the probability of finding the particle within a circle with radius *r* at a given time lag *τ*^52^. First a probability density function (PDF) was created in MATLAB^®^ from the experimental step sizes, and, given the localization accuracy of our measurements, excluding step sizes smaller than 16 nm. The PDF was normalized resulting in the CPD, to which the cumulative probability distribution function (CPF) (Eq. 5) was fitted. The CPF is given in Eq. (5,5), where *D* is the lateral diffusion coefficient and *σ* the localization accuracy.

5 $$\mathrm{CPF}\left( {r^2,\tau } \right) = 1 - \mathrm{e}^{\left( {\frac{{ - r^2}}{{4{{D}}\tau + 4\sigma ^2}}} \right)}$$

The localization accuracy *σ* was determined from the mean error in the x and y parameters of the Gaussian fit. For a homogeneous population of diffusing particles, the cumulative probability function will result in a single exponential decay, while for a heterogeneous diffusive population, the corresponding CPF is expected to resemble the sum of multiple exponentials, depending on the number of populations with a distinct diffusion coefficient. The CPF goodness-of-fit was determined by calculating the residual sum of squares (RSS); the multi-component model that fitted best, i.e., RSS closed to 0, was used to calculate the diffusion coefficient for each population.

### Data availability

Source data for this study are available from the authors upon request.

## Electronic supplementary material

Supplementary Information
