# Supplementary material for: Steric exclusion and protein conformation determine the localization of plasma membrane transporters
Source: Nat Commun. 2018 Feb 5;9:501. doi: 10.1038/s41467-018-02864-2 (PMC5799302; doi:10.1038/s41467-018-02864-2)
Supplement: Supplementary file 1 — Supplementary Information [file 41467_2018_2864_MOESM1_ESM.pdf]

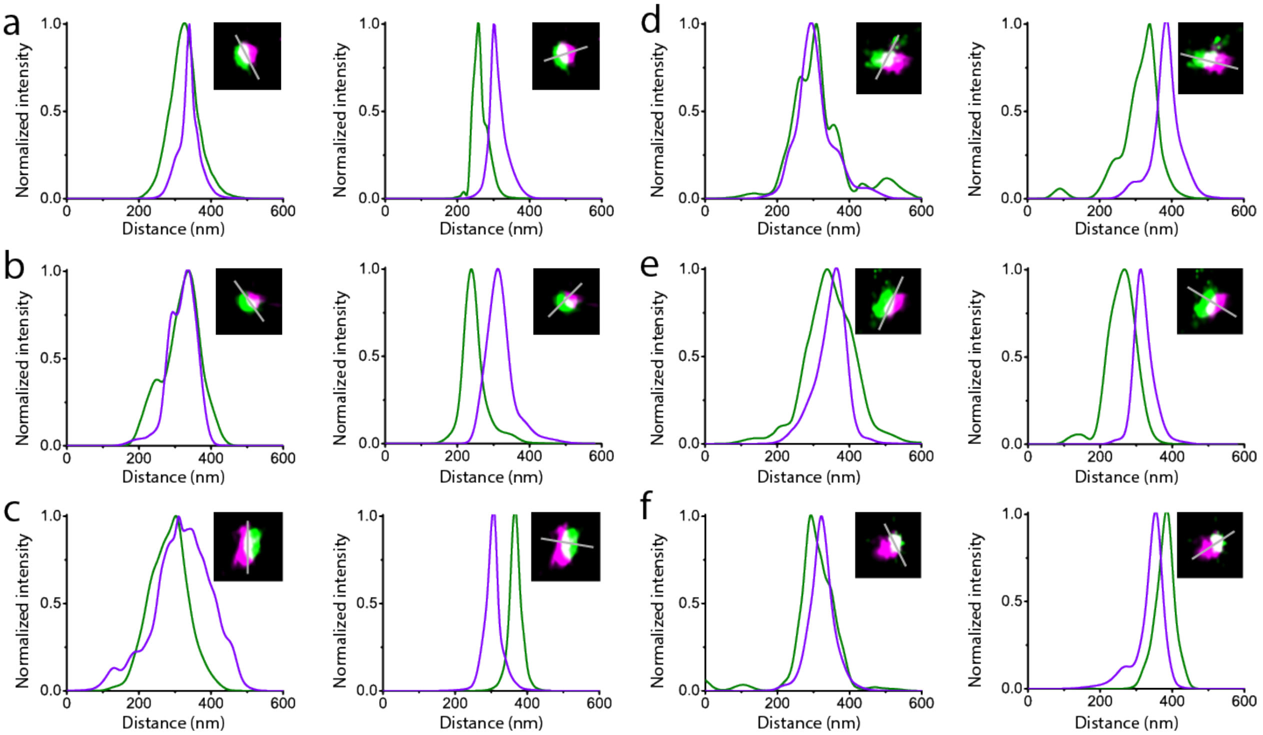

Supplementary Figure 1: Eisosome line-scans measured along and perpendicular to the plasma membrane. Several examples of co-localization of Sur7-YPet in green and Pil1-mKate2 in magenta (panels a to f) to demonstrate that the separation between the two proteins is independent of the orientation of the MCC/eisosome with respect to the x/y axis in the sample plane. Proteins were chromosomally-tagged with the respective fluorophores.

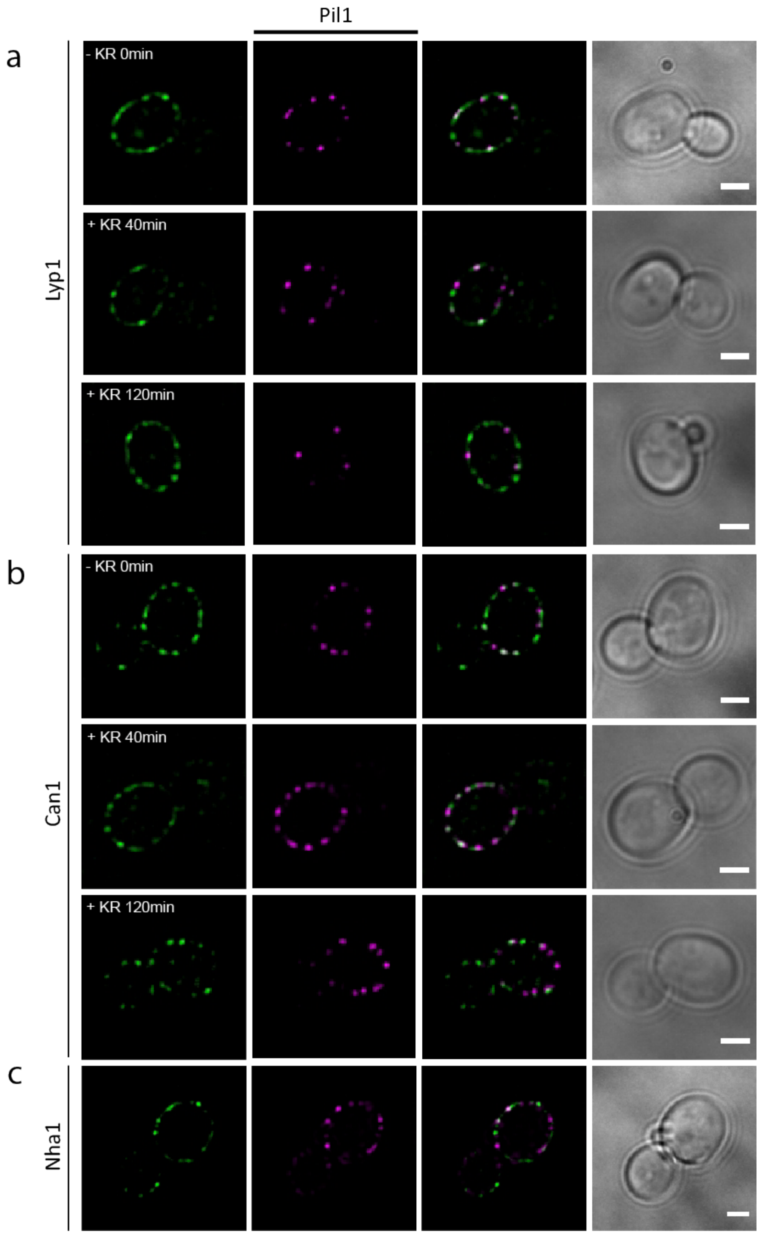

Supplementary Figure 2: Cross-correlation analysis: membrane protein *versus* eisosome reporter. Cross-correlation analysis of Pil1-mKate2 with (a) Lyp1-L-YPet; (b) Can1-L-YPet; and (c) Nha1-L-YPet. The images were treated with a discoidal-averaging filter to illustrate better the localizations; the co-localization analysis was done with the raw diffraction-limited images. Wide-field images are depicted for clarity. All scale bars represent 2 µm. All proteins were chromosomally tagged with the respective fluorophores.

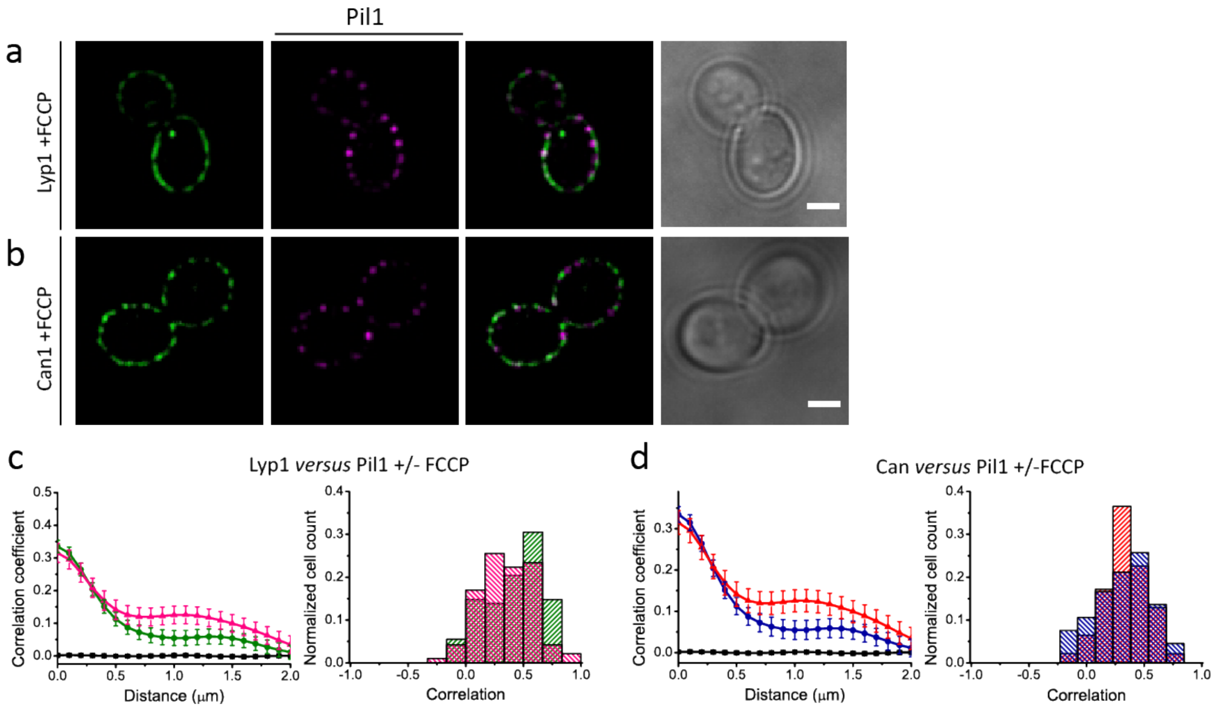

Supplementary Figure 3: Cross-correlation analysis in the presence of protonophore. Dual-colour reconstructions of (a) Lyp1-L-YPet/Pil1-mKate2 and (b) Can1-YPet/Pil1mKate2 upon the addition of the protonophore carbonyl cyanide 4-(trifluoromethoxy)phenylhydrazone (FCCP). *Left panels:* images exemplifying one cell per strain (green: Lyp1 or Can1 fused to YPet; purple: Pil1 fused to mKate2); images were treated with a discoidal-averaging filter to illustrate better the localizations; the co-localization analysis was done with the raw diffraction-limited images. Wide-field images are depicted for clarity. All scale bars represent 2 μm. Cross-correlation of Pil1-mKate2 and YPet-tagged target membrane protein with and without addition of 10 μM FCCP: (c) Lyp1 (n = 94) (d) Can1 (n = 66). *Left panels:* Correlation coefficients of the plasma membrane proteins with FCCP (blue line) without (red line) and the randomized data (black line); errors represent the standard errors of the mean. *Right panels:* normalized heterogeneity in the cross correlation data of single cells treated with FCCP (blue striped bars) or without FCCP (red striped bars). All proteins were expressed from their native chromosomally locations; n represents number of cells analyzed and error bars represent the standard deviation.

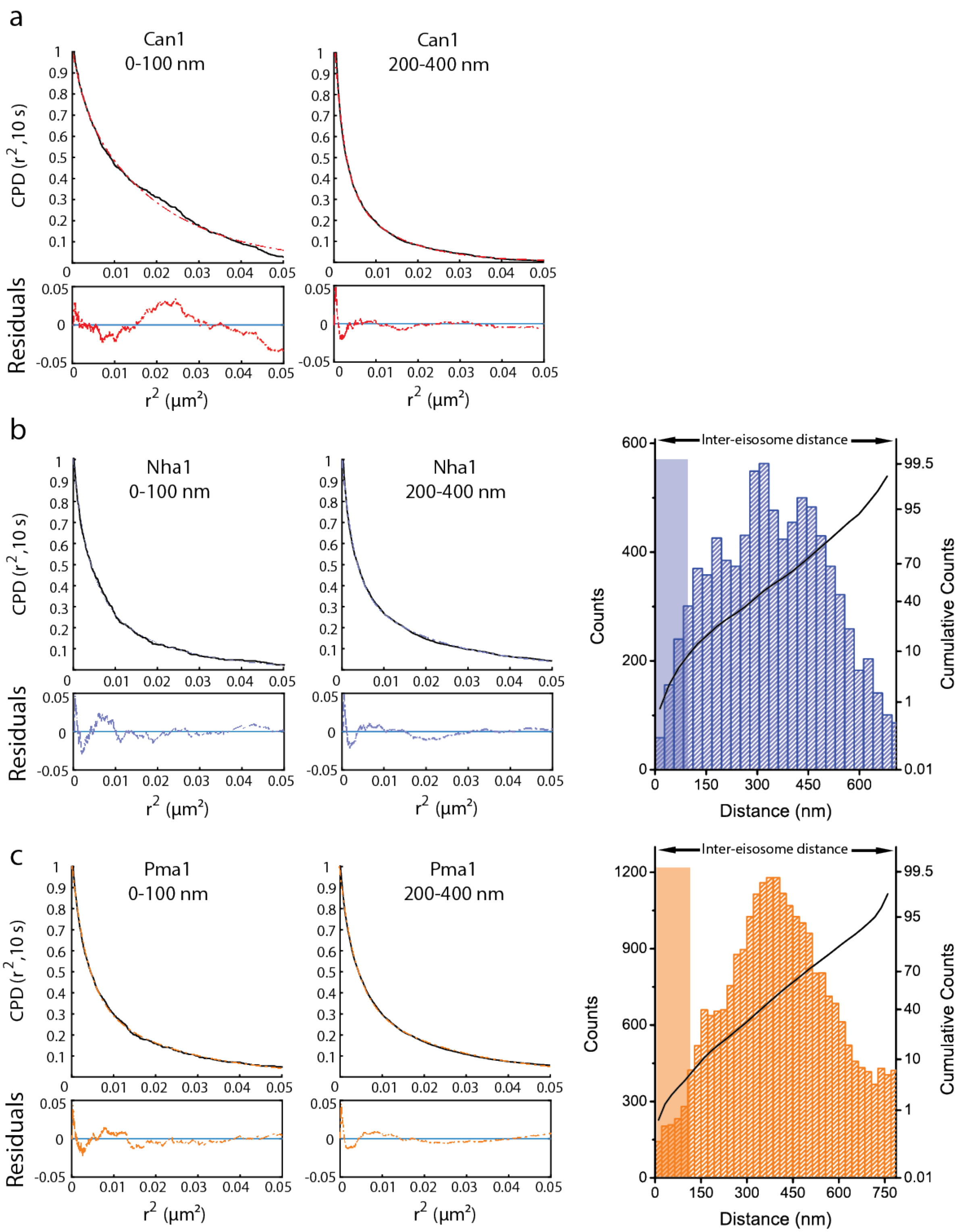

Supplementary Figure 4: Lateral diffusion in relation to distance of Nha1 and Pma1 relative to MCC/eisosomes. Left graphs represent CPD analysis of protein diffusion within the eisosomal area (0-100 nm from the centroid of MCC/eisosome), the middle graphs show the diffusion in the MCP area

(200-400 nm) for Can1 (n = 35) (a), Nha1 (n = 52) (b), and Pma1 (n = 129) (c). Immobile fractions show up as systematic deviations in the fits of the data, which is best seen in the plots of the residuals; *e.g.* the Can1 data at 200-400 nm fit better to the exponential function than those at 0-100 nm. The right graphs of panel b and c show histograms representing localizations of proteins relative to the closest eisosome. Black lines show the probability of finding Nha1 (b) and Pma1 (c) at a specific distance from a MCC/eisosome. The width of the shaded bar on the left of the graphs approximates the length of the MCC/eisosomes.

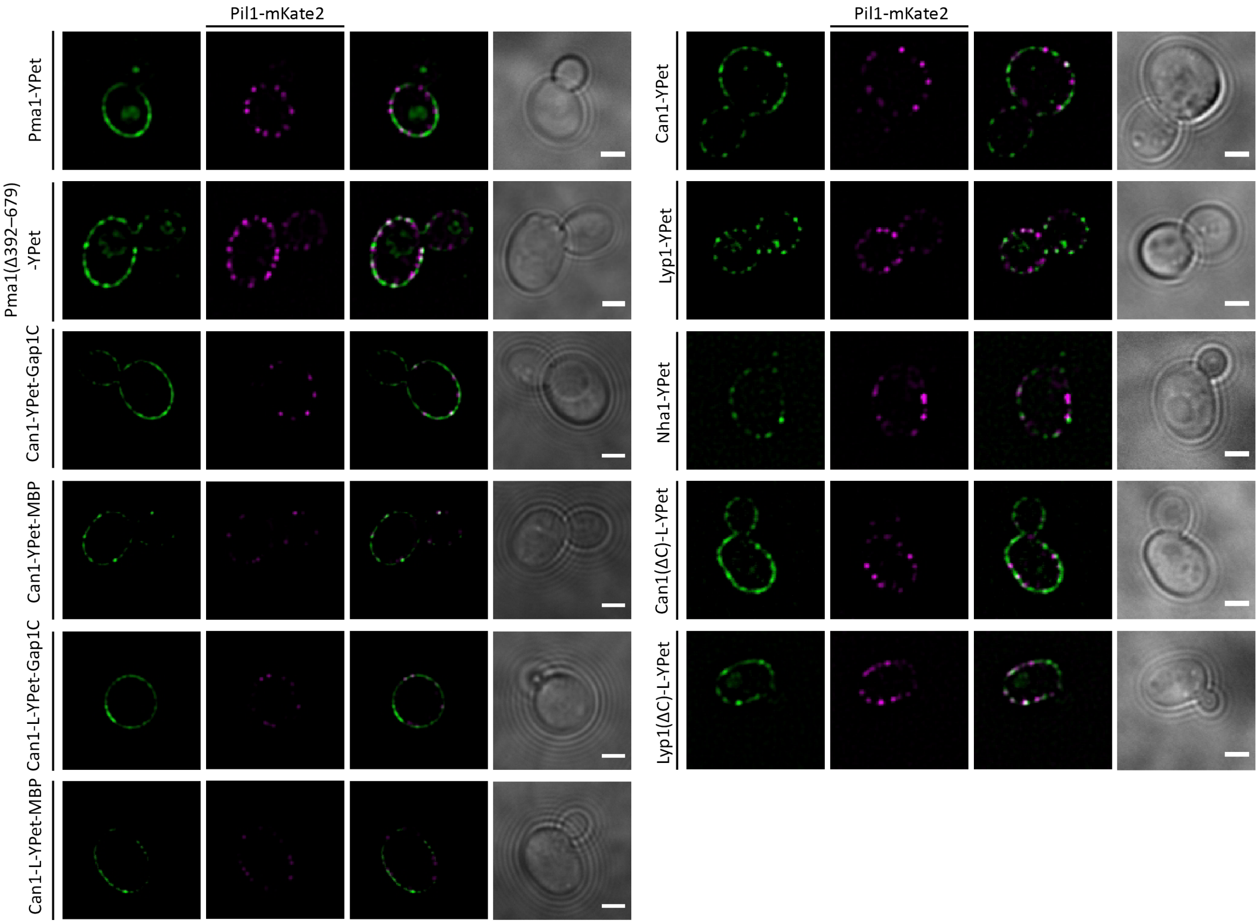

Supplementary Figure 5: Cross-correlation analysis: membrane proteins *w/wo* linker *versus* Pil1. Eisosomal Pil1-mKate2 *versus* Pma1, Can1, Lyp1 and Nha1 variants with reduced linker lengths, or modifications of the proteins as indicated on the left side of the panels. The images show one cell for each strain and condition; images were treated with a discoidal-averaging filter to illustrate better the localizations; the co-localization analysis was done with the raw diffraction-limited images. Wide-field images are depicted for clarity. All scale bars represent 2 µm. All proteins were expressed from their native chromosomal location.

Supplementary Table 1. *Strains used in this study*

**Strains**	**Characteristics**	**Reference**
S. cerevisiae BY4742	*Matα his3Δ1 leu2Δ0 lys2Δ0 ura3Δ0*	Brachmann *et al.* [1]
E. coli MC1061		Casadaban *et al.* [2]
S. cerevisiae BY4742 *Δlyp1*	*Matα his3Δ1 leu2Δ0 lys2Δ0 ura3Δ0 Δlyp1 kanMx*	Giaever *et al.* [3]
S. cerevisiae BY4742 *Δcan1*	*Matα his3Δ1 leu2Δ0 lys2Δ0 ura3Δ0 Δcan1 kanMx*	Giaever *et al.* [3]
S. cerevisiae BY4742 *Δnha1*	*Matα his3Δ1 leu2Δ0 lys2Δ0 ura3Δ0 Δnha1 kanMx*	Giaever *et al.* [3]
S. cerevisiae BY4742 *can1-mEos3.1*	*Matα his3Δ1 leu2Δ0 lys2Δ0 can1::CAN1-MEOS3.1_URA3*	This study
S. cerevisiae BY4742 *lyp1-mEos3.1*	*Matα his3Δ1 leu2Δ0 lys2Δ0 lyp1::LYP1-MEOS3.1_URA3*	This study
S. cerevisiae BY4742 *lyp1-YPet*	*Matα his3Δ1 leu2Δ0 lys2Δ0 ura3Δ0 lyp1::LYP1-YPET*	This Study
S. cerevisiae BY4742 *can1-YPet*	*Matα his3Δ1 leu2Δ0 lys2Δ0 ura3Δ0 can1::CAN1-YPET*	This Study
S. cerevisiae BY4742 *pil1-mKate2*	*Matα his3Δ1 leu2Δ0 lys2Δ0 ura3Δ0 pil1::PIL1-MKATE2*	This Study
S. cerevisiae BY4742 *lyp1-YPet pil1-mKate2*	*Matα his3Δ1 leu2Δ0 lys2Δ0 ura3Δ0 lyp1::LYP1-YPET_URA3 pil1::PIL-MKATE2*	This Study
S. cerevisiae BY4742 *lyp1-L-YPet pil1-mKate2*	*Matα his3Δ1 leu2Δ0 lys2Δ0 ura3Δ0 lyp1::LYP1-L-YPET_URA3 pil1::PIL-MKATE2*	This Study
S. cerevisiae BY4742 *lyp1(ΔC)-L-YPet pil1-mKate2*	*Matα his3Δ1 leu2Δ0 lys2Δ0 ura3Δ0 lyp1::LYP1(Δ601-611)-L-YPET_URA3 pil1::PIL-MKATE2*	This Study
S. cerevisiae BY4742 *can1-YPet pil1-mKate2*	*Matα his3Δ1 leu2Δ0 lys2Δ0 ura3Δ0 can1::CAN1-YPET_URA3 pil1::PIL1-MKATE2*	This Study
S. cerevisiae BY4742 *can1-L-YPet pil1-mKate2*	*Matα his3Δ1 leu2Δ0 lys2Δ0 ura3Δ0 can1::CAN1-L-YPET_URA3 pil1::PIL1-MKATE2*	This Study
S. cerevisiae BY4742 *can1(ΔC)-L-YPet pil1-mKate2*	*Matα his3Δ1 leu2Δ0 lys2Δ0 ura3Δ0 ura3Δ0 can1::CAN1(Δ580-590)-L-YPET_URA3 pil1::PIL1-MKATE2*	This Study
S. cerevisiae BY4742 *nha1-YPet pil1-mKate2*	*Matα his3Δ1 leu2Δ0 lys2Δ0 ura3Δ0 nha1::NHA1-YPET_URA3 pil1::PIL1-MKATE2*	This Study
S. cerevisiae BY4742 *nha1-L-YPet pil1-mKate2*	*Matα his3Δ1 leu2Δ0 lys2Δ0 ura3Δ0 nha1::NHA1-L-YPET_URA3 pil1::PIL1-MKATE2*	This Study
S. cerevisiae BY4742 *sur7-YPet pil1-mKate2*	*Matα his3Δ1 leu2Δ0 lys2Δ0 ura3Δ0 sur7::SUR7-YPET pil1::PIL1-MKATE2_URA3*	This Study
S. cerevisiae BY4742 *can1-YPet-MBP pil1-mKate2*	*Matα his3Δ1 leu2Δ0 lys2Δ0 ura3Δ0 can1::CAN1-YPET-MBP_HIS3 pil1::PIL1-MKATE2*	This Study
S. cerevisiae BY4742 *can1-L-YPet-MBP pil1-mKate2*	*Matα his3Δ1 leu2Δ0 lys2Δ0 ura3Δ0 can1::CAN1-L-Ypet-MBP_HIS3 pil1::PIL1-MKATE2*	This Study
S. cerevisiae BY4742 can-YPet-gapC pil1-mKate2	*Mat α his3 Δ 1 leu2 Δ 0 lys2 Δ 0 ura3 Δ 0 can1::CAN1-YPET-GAPC_LEU2 pil1::PIL1-MKATE2*	This Study
S. cerevisiae BY4742 can-L-YPet-gapC	*Mat α his3 Δ 1 leu2 Δ 0 lys2 Δ 0 ura3 Δ 0 can1::CAN1-YPET-GAPC_LEU2 pil1::PIL1-MKATE2*	This Study
S. cerevisiae BY4742 *sur7-YPet*	*Matα his3Δ1 leu2Δ0 lys2Δ0 ura3Δ0 sur7::SUR7-YPET*	This Study
S. cerevisiae BY4742 can-L-mCardinal sur7-YPet	*Matα his3Δ1 leu2Δ0 lys2Δ0 ura3Δ0 sur7::SUR7-YPET can1::CAN1-MCARDINAL*	This Study

Supplementary Table 2. *Plasmids used in this study*

**Plasmids**	**Characteristics**	**Reference**
pRS426GAL1-GFP	pRS426 with *gal* promoter and GFP-His fusion cassette with *ura3* selection marker	Newstead *et al.*[4]
pRS316	Centromeric shuttle vector with *ura3* marker	Sikorski *et al.*[5]
pRS317	Centromeric shuttle vector with *lys2* marker	Sikorski *et al.*[5]
pFB001	pRS426GAL1-GFP derivative with *lyp1* fused to *YPet-his*	This study
pFB002	pRS426GAL1-GFP derivative with *can1* fused to *YPet-his*	This study
pFB003	pRS426GAL1-GFP derivative with *nha1* fused to *YPet-his*	This study
pFB004	pRS426GAL1-GFP derivative with *vba1* fused to *YPet-his*	This study
pUG72	Amp^{R}, *Ura3* chromosomal integration cassette	Gueldener *et al.* [6]
pFB007	Pug 72 with *ura3* flanking homologous regions and reverse *mEos3.1* fusion gene	This Study
pFB008	Pug 72 with *ura3* flanking homologous regions and reverse *YPet* fusion gene	This Study
pFB009	Pug 72 with *ura3* flanking homologous regions and reverse *mKate2* fusion gene	This Study

pLS001	pRS316 derivative with Pma1-YPet under Pma1 promoter	This Study
pLS002	pLS001 dertivative with Pma1(Δ392–679)-YPet	This Study
pLS003	pRS426GAL1-GFP derivative with *can1* fused to *YPet with linker*	This Study
pLS004	pRS426GAL1-GFP derivative with *lyp1* fused to *YPet with linker*	This Study
pLS005	pRS426GAL1-GFP derivative with *can1* fused to *mCardinal with linker*	This Study
pPP002	Adapted from pACM040, MBP-GFP under GAL1 promoter	Popken *et al.* [7]
pUG73	*Amp*^{R}*, Leu2* chromosomal integration cassette	Gueldener *et al.* [6]
pLS006	Pug 73 with *leu2* and reverse *gap1C* fusion gene	This Study
pDP001-GFP-Gap1C	pACM021-GFP with Gap1(552-602) fused to GFP	Popov et al [8]
pLS007	pRS426GAL1-GFP derivative with *nha1* fused to *mCardinal with linker*	This Study
pLS008	pRS426GAL1-GFP derivative with *pma1* fused to *mCardinal with linker*	This study

Supplementary Table 3. *Primers used in this study* (italics indicates homologous regions introduced for the excision of the *Ura3* marker after genomic integration of the Pug72 *mEos3.1* cassette)

Primer name	Sequence	Purpose
Pr1	ACCACCACCAUCATCATCATCATTAACTGCAGGAATTC	Fw primer for amplification of pRS426GAL1-GFP vector annealing at histag for swaping c terminal fusion protein.
Pr2	AGGGTAGTGCUGAAGGAAGCATACGATACCC	Fw primer for amplification of pRS426GAL1-GFP
Pr3	AGCACTACCCUTTAGCTGTTCTATATGCTGCC	Rev primer for amplification of pRS426GAL1-GFP
Pr4	ATTTTGGGAUCCACTAGTTCTAGAATCCGGGG	Rev primer for pRS426GAL1-GFP backbone amplification anneals behind *gal* promoter.
Pr5	AGGGGAAAAUTTATATTTTCAAGGTTCTAAAGGTGAAGAATTATTCA CTGG	Fw primer for amplification of *YPet gene* and insertion into pRS426GAL1-GFP.
Pr6	ATGGTGGTGGUGGAGCTCTTTGTACAATTCATTCATACC	Rev primer for amplification of *YPet gene* and insertion into pRS426GAL1-GFP.
Pr7	ATCCCAAAAUGGGCAGGTTTAGTAACATAATAACGTCC	Fw primer for amplification of *S. cerevisiae lyp1* gene for insertion into pRS426GAL1-GFP.
Pr8	ATTTTCCCCUCCTGCAACAGCAGCCCAGAATTTCTC	Rev primer for amplification of *S. cerevisiae lyp1* gene for insertion into pRS426GAL1-GFP.
Pr9	ATTTTCCCCUCCTGCTACAACATTCCAAAATTTGTCCC	Fw primer for amplification of *S. cerevisiae can1* gene for insertion into pRS426GAL1-GFP.
Pr10	ATCCCAAAAUGGGAACAAATTCAAAAGAAGACGCCGACATAG	Rev primer for amplification of *S. cerevisiae can1* gene for insertion into pRS426GAL1-GFP.
Pr11	ATCCCAAAAUGGCTATCTGGGAGCAACTAGAAG	Fw primer for amplification of *S. cerevisiae nha1* gene for insertion into pRS426GAL1-GFP.
Pr12	ATTTTCCCCUCCCTTATTGAGACCAAGCGTTTTTGATAGCG	Rev primer for amplification of *S. cerevisiae nha1* gene for insertion into pRS426GAL1-GFP.
Pr13	ATCCCAAAAUGGGACAAACACTAGACGAGACTTCAAATCTAC	Fw primer for amplification of *S. cerevisiae vba1* gene for insertion into pRS426GAL1-GFP.
Pr14	ATTTTCCCCUCCAGAACTTGAACTACGTTTGTAAGTATGTTTC	Rev primer for amplification of *S. cerevisiae vba1* gene for insertion into pRS426GAL1-GFP.

Pr19	AGTCCGACCU*GGGCGCGCGCGAATTAGCCGCGGCGTGGCCTTCCCA AATTTGGGTAGGG*CGTCTAGAGATCCCAATACAACAGATCAC	Primer for construction of the pug72 *mEos3.1* fusion cassette, containing upstream homologous region flanking *Ura3*, for excision of the *Ura3* marker after genomical integration.
Pr20	ACTAACCCGU*GGGCGCGCGCGAATTAGCCGCGGCGTGGCCTTCCCA AATTTGGGTAGGGC*CTCGAGAACCCTTAATATAACTTCGT	Primer for construction of the pug72 *mEos3.1* fusion cassette, containing downstream homologous region flanking *Ura3*, for excision of the *Ura3* marker after genomical integration.
Pr21	ACGGGAACGUCGTACGAAGCTTCAGCTGGC	Rev primer for amplification of Pug72 backbone for insertion of fluorescent protein in pug72 vector.
Pr22	ACGGGTTAGUAGCTCGTTTTATTTAGGTTCTATCGAGG	*Rev primer for amplification of Ura3* marker for Pug72 *mEos3.1* cassette.
Pr23	ACGTTCCCGUATGAGTGCGATTAAGCCAGACA	Fw primer for Amplification of *mEos3.1* for pug72 *mEos3.1* fusion cassette
Pr24	AGGTCGGACUTTATCGTCTGGCATTGTCAGGC	Rev primer for Amplification of *mEos3.1* for pug72 *mEos3.1* fusion cassette
Pr25	ACGTTCCCGUATGTCTAAAGGTGAAGAATTATTCACTGG	Fw primer for Amplification of *YPet* for pug72 *YPet* fusion cassette
Pr26	AGGTCGGACUTTAGAGCTCTTTGTACAATTCATTCATAC	Rev primer for Amplification of *YPet* for pug72 *YPet* fusion cassette
Pr27	ACGTTCCCGUATGGTGAGCGAGCTGATTAAGG	Fw primer for Amplification of *mKate2* for pug72 *mKate2* fusion cassette
Pr28	AGGTCGGACUTCATCTGTGCCCCAGTTTGCTAG	Rev primer for Amplification of *mKate2* for pug72 *mKate2* fusion cassette
Pr29	GCGAAATGGCGTGGAAATGTGATCAAAGGTAATAAAACGTCATATC TGATATCACCTAATAACTTCG	Rev primer for amplification of *can1* specific fusion cassette from Pug72
Pr30	GAAGATCATGAACCAAAGACTTTTTGGGACAAATTTTGGAATGTTGT AGCAATGAGTGCGATTAAGCCAGAC	Fw primer for amplification of *can1* specific *mEos3.1* fusion cassette from Pug72 *mEos3.1*
Pr31	GAAGATCATGAACCAAAGACTTTTTGGGACAAATTTTGGAATGTTGT AGCAATGTCTAAAGGTGAAGAATTATTCACTGG	Fw primer for amplification of *can1* specific *YPet* fusion cassette from Pug72 *YPet*
Pr32	TGAACCAAAGACTTTTTGGGACAAATTTTGGAATGTTGTAGCACGTA CGCTGCAGGTCGACGGAGCAGGTGCTGGTGCTGGTGCTGGAGCAA TGTCTAAAGGTGAAGAATTATTCACTGG	Fw primer for amplification of *can1* specific *YPet* fusion cassette from Pug72 *YPet* with linker
Pr33	AGACATTGAGGCAATTGTATGGGAAGATCATGAACCAAAGACTCGT ACGCTGCAGGTCGACGGAGCAGGTGCTGGTGCTGGTGCTGGAGCA ATGTCTAAAGGTGAAGAATTATTCACTGG	Fw primer for amplification of *can1Δ10* specific *YPet* fusion cassette from Pug72 *YPet* with linker
Pr34	CTATTTTTTTATTTTTTTCTATTTTGAAGGCATGCAAGAGGTTCTGTGA CTGATATCACCTAATAACTTCG	Rev primer for amplification of *lyp1* specific fusion cassette from Pug72
Pr35	GAAGACGACGAGCCTAAGAATTTATGGGAGAAATTCTGGGCTGCTG TTGCAATGAGTGCGATTAAGCCAGAC	Fw primer for amplification of *lyp1* specific *mEos3.1* fusion cassette from Pug72 *mEos3.1*
Pr36	GAAGACGACGAGCCTAAGAATTTATGGGAGAAATTCTGGGCTGCTG TTGCAATGTCTAAAGGTGAAGAATTATTCACTGG	Fw primer for amplification of *lyp1* specific *YPet* fusion cassette from Pug72 *YPet.*
Pr36	CGAGCCTAAGAATTTATGGGAGAAATTCTGGGCTGCTGTTGCACGT ACGCTGCAGGTCGACGGAGCAGGTGCTGGTGCTGGTGCTGGAGCA ATGTCTAAAGGTGAAGAATTATTCACTGG	Fw primer for amplification of *lyp1* specific *YPet* fusion cassette from Pug72 *YPet* with linker
Pr37	AGAAATCGAAGCAATTATTTGGGAAGACGACGAGCCTAAGAATCGT ACGCTGCAGGTCGACGGAGCAGGTGCTGGTGCTGGTGCTGGAGCA ATGTCTAAAGGTGAAGAATTATTCACTGG	Fw primer for amplification of *lyp1Δ10* specific *YPet* fusion cassette from Pug72 *YPet* with linker
Pr38	CATTTCGTTTATATATATACTAAAATAATATATCTTTGTGTATTAATAA ATGGATCTGATATCACCTAATAACTTCG	Rev primer for amplification of *nha1* specific fusion cassette from Pug72
Pr39	GAGTGCTGCTGTTAAGTCGGCGCTATCAAAAACGCTTGGTCTCAATA AGGGAGGGGGAATGTCTAAAGGTGAAGAATTATTCACTGG	Fw primer for amplification of *nha1* specific *YPet* fusion cassette from Pug72 *YPet*
Pr40	TGCTGTTAAGTCGGCGCTATCAAAAACGCTTGGTCTCAATAAGCGTA CGCTGCAGGTCGACGGAGCAGGTGCTGGTGCTGGTGCTGGAGCAA TGTCTAAAGGTGAAGAATTATTCACTGG	Fw primer for amplification of *nha1* specific *YPet* fusion cassette from Pug72 *YPet* with linker
Pr41	CATGAACAAGTCGGACACCAGCAAAGTGAGTCTCTTCCCCAACAAAC AACAGCTGGAGGGGGAATGGTGAGCGAGCTGATTAAGG	Fw primer for amplification of *pil1* specific *mKate2* fusion cassette from Pug72 *mKate2*
Pr42	CTGCTGGTTTTTTTTTTTTTTGTTTCTAATAGATTGTTGATTTATTTTGA ATCTGATATCACCTAATAACTTCG	Rev primer for amplification of *pil1* specific fusion cassette from Pug72
Pr43	CTTCTTCACTATAAGAAAATCACACGAGCGCCCGGACGATGTCTCTG TTGGAGGTGGAATGTCTAAAGGTGAAGAATTATTCACTGG	Fw primer for amplification of *sur7* specific *YPet* fusion cassette from Pug72 *YPet.*
Pr44	GAGAAGAAAGGGGTATAAATATATATTACAAAGCGGAAAACTTGCG CCATGGATCTGATATCACCTAATAACTTCG	Rev primer for amplification of *sur7* specific fusion cassette from Pug72
Pr45	CACCGCGGTGGCGGCCGCTCTAGAACTAGTGGATCCCCCAAAACAA	Forward primer for cloning Pma1 with its

	ACCCGGTCTCGAAG	promoter from *S. cerevisiae* chromosome 7 to prs316 using homologous recombination
Pr46	AATAATTCTTCACCTTTAGAACCCCCTCCGCCACCGGTTTCCTTTTCGTGTTGAGTAGAG	Rev primer for cloning Pma1 from *S. cerevisiae* chromosome 7 and homologous recombination with YPet fw
Pr47	TCAACACGAAAAGGAAACCGGTGGCGGAGGGGGTTCTAAAGGTGAAGAATTATTCACTGGTGTTGTCC	Fw primer for cloning YPet and fusing to Pma1
Pr48	GACGGTATCGATAAGCTTGATATCGAATTCCTGCAGCCCTTAGAGCTCTTTGTACAATTCATTCATACCCTCG	Rev primer for cloning YPet and fusing to Pma1
Pr49	AGAACCACCAGCACCACCAGAAGAAGCAGCACCTCCAGACAAATTTTTCACAGAATGTACTCT	Forward primer for removal of cytosolic part of Pma1. Anneals at T680. contains homologous region with Pr44
Pr50	GCTGCTTCTTCTGGTGGTGCTGGTGGTTCTTTCGTGCAAGGACAACTTGTTCTTGGTCAA	Reverse primer for removal of cytosolic part of Pma1. Finishes at E392. contains homologous region with Pr43
Pr51	ATCCCAAAAUGGGAACAAATTCAAAAGAAGACGCCGACATAG	Fw primer for amplification of *S. cerevisiae lyp1-L-YPet* insertion into pRS426GAL1-GFP.
Pr52	ATCCCAAAAUGGGAACAAATTCAAAAGAAGACGCCGACATAG	Fw primer for amplification of *S. cerevisiae can1-L-YPet* insertion into pRS426GAL1-GFP.
Pr53	ATGGTGGTGGUGGAGCTCTTATTTGTACAATTCATTCATACC	Rev primer for amplification of *S. cerevisiae YPet gene* insertion into pRS426GAL1-GFP.
Pr54	TGCTACAACATTCCAAAATTTGTCC	Fw primer for amplification of pLS003 without the fluorescent protein
Pr55	TCATCATCATCATTAACTGCAGG	Rev primer for amplification of pLS003 without the fluorescent protein
Pr56	GAAGATCATGAACCAAAGACTTTTTGGGACAAATTTTGGAATGTTGTAGCACGTACGCTGCAGGTCGACGGAGCAGGTGCTGGTGCTGGTGCTGGAGCAATGGTCAGTAAGGGTGAAGAA	Fw primer for amplifying mCardinal with regions homologous to pLS003 plasmid
Pr57	GATAAGCTTGATATCGAATTCCTGCAGTTAATGATGATGATGATGGAGCTCTTATTACTTGTATAATTCGTCCATACCATTC	Rev primer for amplifying mCardinal with regions homologous to pLS003 plasmid
Pr58	AATTTTTGACTGCTGCTGGTATTACCGAGGGTATGAATGAATTGTACAAATCTAGAACTAGTGGATCCCCCGGG	Fw primer for amplification of *YPet* specific fusion cassette from Pug73 gapC
Pr59	AGAATGCGAAATGGCGTGGAAATGTGATCAAAGGTAATAAAACGTCATCTCACTATAGGGAGACCG	Rev primer for amplification of *can1* specific fusion cassette from Pug73 gapC
Pr60	AATTTTTGACTGCTGCTGGTATTACCGAGGGTATGAATGAATTGTACAAAGC	Fw primer for amplification of *YPet* specific fusion cassette from pPP002
Pr61	AGAATGCGAAATGGCGTGGAAATGTGATCAAAGGTAATAAAACGTCATATCGAGGCCCTTTCGT	Rev primer for amplification of *can1* specific fusion cassette from pPP002
Pr62	AATGTGTAAUGGCCTTGAGATTTCAAGC	Fw primer to amplify pUG73 backbone
Pr63	AACGACAATAUGTCCATATGGTG	Rev primer to amplify pUG73 backbone
Pr64	ATATTGTCGTUGTACAAATCTAGAACTAGTGGATCC	Fw primer to amplify *gapC* coding region
Pr65	ATTACACATUTCACTATAGGGCGAATTGGGTAC	Rev primer to amplify *gapC* coding region
Pr66	ATTTTGGGATCCACTAGTTCTAGAATCCGG	Fw primer to amplify first part of the backbone
Pr67	TGCACTCTCAGTACAATCTGCTC	Rev primer to amplify first part of the backbone
Pr68	CGTACGCTGCAGGTCGACG	Fw primer to amplify second part of the backbone
Pr69	GGCTTAACTATGCGGCATC	Rev primer to amplify second part of the backbone
Pr70	CTTTAACGTCAAGGAGAAAAAACCCCGGATTCTAGAACTAGTGGATCCCAAAATATGGCTATCTGGGAGCAACTA	Fw primer to amplify *nha1* gene
Pr71	CATTGCTCCAGCACCAGCACCAGCACCTGCTCCGTCGACCTGCAGCGTACGCTTATTGAGACCAAGCGTTTTTGATA	Rev primer to amplify *nha1* gene
Pr72	AATATACCTCTATACTTTAACGTCAAGGAGAAAAAACCCCGGATTCTAGAATGACTGATACATCATCCTCTTCA	Fw primer to amplify *pma1* gene
Pr73	AGCACCTGCTCCGTCGACCTGCAGCGTACGGGTTTCCTTTTCGTGTTG	Rev primer to amplify *pma1* gene

1. Brachmann, C. B. *et al.* Designer deletion strains derived from *Saccharomyces cerevisiae* S288C: a useful set of strains and plasmids for PCR-mediated gene disruption and other applications. *Yeast* **14,** 115–132 (1998).
2. Casadaban, M. J. & Cohen, S. N. Analysis of gene control signals by DNA fusion and cloning in Escherichia coli. *Journal of Molecular Biology* **138,** 179–207 (1980).
3. Giaever, G. *et al.* Functional profiling of the *Saccharomyces cerevisiae* genome. *Nature* **418,** 387–391 (2002).
4. Newstead, S. *et al.* High-throughput fluorescent-based optimization of eukaryotic membrane protein overexpression and purification in Saccharomyces cerevisiae. *Proc Natl Acad Sci U S A* **104,** 13936–13941 (2007).
5. Sikorski, R. S. & Hieter, P. A system of shuttle vectors and yeast host strains designed for efficient manipulation of DNA in Saccharomyces cerevisiae. *Genetics* **122,** 19–27 (1989).
6. Gueldener, U., Heinisch, J., Koehler, G. J., Voss, D. & Hegemann, J. H. A second set of loxP marker cassettes for Cre-mediated multiple gene knockouts in budding yeast. *Nucleic Acids Res.* **30,** e23 (2002).
7. Popken, P., Ghavami, A., Onck, P. R., Poolman, B. & Veenhoff, L. M. Size-dependent leak of soluble and membrane proteins through the yeast nuclear pore complex. *Mol. Biol. Cell* **26,** 1386–1394 (2015).
8. Popov-Čeleketić, D., Bianchi, F., Ruiz, S. J., Meutiawati, F. & Poolman, B. A Plasma Membrane Association Module in Yeast Amino Acid Transporters. *Journal of Biological Chemistry* **291,** 16024–16037 (2016).
